# Disaggregation of Amylin Aggregate by Novel Conformationally Restricted Aminobenzoic Acid containing α/β and α/γ Hybrid Peptidomimetics

**DOI:** 10.1038/srep40095

**Published:** 2017-01-05

**Authors:** Ashim Paul, Sourav Kalita, Sujan Kalita, Piruthivi Sukumar, Bhubaneswar Mandal

**Affiliations:** 1 Laboratory of Peptide and Amyloid Research, Department of Chemistry, Indian Institute of Technology Guwahati, Assam- 781039, India; 2 Department of Biosciences and Bioengineering, Indian Institute of Technology Guwahati, Assam-781039, India; 3 Leeds Institute of Cardiovascular and Metabolic Medicine, LIGHT Laboratories, University of Leeds, Leeds LS2 9JT, UK

## Abstract

Diabetes has emerged as a threat to the current world. More than ninety five per cent of all the diabetic population has type 2 diabetes mellitus (T2DM). Aggregates of Amylin hormone, which is co-secreted with insulin from the pancreatic β-cells, inhibit the activities of insulin and glucagon and cause T2DM. Importance of the conformationally restricted peptides for drug design against T2DM has been invigorated by recent FDA approval of Symlin, which is a large conformationally restricted peptide. However, Symlin still has some issues including solubility, oral bioavailability and cost of preparation. Herein, we introduced a novel strategy for conformationally restricted peptide design adopting a minimalistic approach for cost reduction. We have demonstrated efficient inhibition of amyloid formation of Amylin and its disruption by a novel class of conformationally restricted β-sheet breaker hybrid peptidomimetics (BSBHps). We have inserted β, γ and δ -aminobenzoic acid separately into an amyloidogenic peptide sequence, synthesized α/β, α/γ and α/δ hybrid peptidomimetics, respectively. Interestingly, we observed the aggregation inhibitory efficacy of α/β and α/γ BSBHps, but not of α/δ analogues. They also disrupt existing amyloids into non-toxic forms. Results may be useful for newer drug design against T2DM as well as other amyloidoses and understanding amyloidogenesis.

Protein aggregation and amyloid deposition cause diverse human diseases including Alzheimer’s disease (AD), Parkinson’s disease (PD), and Type-2 Diabetes Mellitus (T2DM)^1^,^2^. Although, amyloid fibrils are generated from different proteins, they exhibit similar properties including insolubility and morphology^3^,^4^,^5^. These amyloid fibrils are comprised of polypeptides organised in a stacked cross-β sheet conformation^3^. Pancreatic amyloid deposits in human, comprised of islet amyloid polypeptide (hIAPP, a 37-amino acid residue polypeptide chain, also known as Amylin), are found in more than 95% of T2DM patients and toxicity caused by them is believed to be a major reason for the pancreatic-β-cell dysfunction and pathogenesis of the diseasse^6^,^7^,^8^. Amylin or hIAPP is a peptide hormone co-secreted with insulin from the pancreatic β-cells of islets of Langerhans. It aggregates from non-toxic native monomers to cytotoxic oligomers at physiological condition and inhibits the activities of glucagon and β-cells leading to cause T2DM^7^,^8^. The non-toxic native monomer of hIAPP misfold to β-sheet rich oligomer which perform membrane fragmentation and destroy the pancreatic β-cells by pore formation^9^,^10^. Therefore, blocking the ability of the amyloidogenic peptide to adopt a β-sheet rich conformation and impairment of the toxic oligomers would be an important strategy to inhibit the amyloid formation and drug development against T2DM and other amyloid related diseases^11^,^12^,^13^,^14^.

The small peptide fragments of hIAPP, hIAPP_22-29_ (NFGAILSS), hIAPP_22-27_ (NFGAIL) and also hIAPP_23-27_ (FGAIL) are known to form amyloid fibrils *in vitro* similar to that of the wild type hIAPP_1-37_^14^,^15^. Thus, targeting the small peptide fragments and designing small peptide based inhibitor comprised of breaker element and recognition segment is accepted as a promising therapeutic approach against amyloid formation and its disruption. Many small peptide based inhibitors with various breaker elements are reported in literature, including proline^16^, α-aminoisobutyric acid (Aib)^17^, α,β-dehydrophenylalanine^18^, *N*-methylated amino acids^19^ and aspartic acid derivative^20^. Among them, the first three types of breaker peptides are designed by the insertion of conformational restriction in it. Among them, the strategy of proline insertion has found great success with the FDA approval of Symlin or Pramlintide. However, not many strategies for inducing conformational restriction have been tested for the design of T2DM related breaker peptides. Moreover, Symlin also has some drawbacks, such as cost of preparation, solubility and oral bioavailability. Therefore, better drugs are always welcome. Since, most of these breaker elements contain α-amino acids; they are proteinogenic, thus unstable against proteolytic degradation. On the other hand, 2-aminobenzoic acids is structurally rigid and favours the formation of helical or turn conformation while present in a peptide sequence^21^,^22^,^23^. Also it is the precursor for the biosynthesis of tryptophan^24^. Furthermore, 2-aminobenzoic acid derivatives have been used as transthyretin amyloid fibril^25^ and amyloid β peptide^26^ aggregation inhibitors. We hypothesized that the hybrid peptides containing β, γ and δ aminobenzoic acids would be more stable towards proteolytic degradation^27^,^28^, and thus a better candidate that inhibit/disrupt hIAPP aggregation. We wanted to find better lead molecules for drug design against T2DM by a minimalistic design approach and incorporation of conformational restriction using differently substituted amino benzoic acids.

We have inserted one or more conformationally restricted aromatic amino acids, β (2-aminobenzoic acid), γ (3-aminobenzoic acid) and δ (4-aminobenzoic acid) separately in the hIAPP_22-27_ peptide sequence, synthesized α/β, α/γ, and α/δ hybrid peptidomimetics, and examined their enzymatic stability and inhibitory effect on the aggregation of the wild type hIAPP_1–37_. We have also investigated the efficacy of the synthesized peptidomimetics for the disruption of hIAPP_1-37_ aggregates and examined whether the disrupted fibrils are toxic soluble oligomers. Inherent toxicity of the selected compounds has been also checked by MTT reduction assay in human embryonic kidney (HEK) cells *in vitro*.

## Results

The wild type hIAPP_1-37_ is an amyloidogenic polypeptide and is prone to form amyloid^16^. Therefore, the development of effective inhibitors against the wild-type hIAPP amyloid formation is a challenging task. To achieve our target, we first synthesiszed the core hydrophobic region of the wild type hIAPP, hIAPP_22-27_ (NFGAIL, peptide **1**), which has a propensity to form amyloid fibrils similar to that of the wild type hIAPP^14^. We designed and synthesized six β-sheet breaker hybrid peptidomimetics (BSBHps) by incorporating various isomers of aminobenzoic acid (2, 3 and 4-aminobenzoic acids, respectively) as breaker element seperately into peptide **1** (Table 1) and later investigated their inhibitory efficacy on the amyloid formation of hIAPP. We also sythesized two control breaker peptides with the incorporation of α-aminoisobutyric acid (Aib)^29^,^30^ into peptide **1** (Table 1).

The different breaker elements were incorporated in all the breaker peptidomimetics keeping the -N-F- dipeptidyl residue same for mentaining the sequence homology with hIAPP_22-23_. The breaker elements were incorporated either at I26 or simultaneously at G24 and I26^31^. All the synthesized peptidomimetics were characterized by HPLC and ESI-MS spectrometry (Figures S1–S18, ESI). We first examined the amyloidogenic nature of these BSBHps and other synthesized peptides by various biophysical tools. The peptidomimetics were dissolved in phosphate buffer solution (PBS, 50 mM) pH 7.4 and incubated at 37 °C for 5 days. The self-aggregation propensity was characterized after 5 days by TEM and Congo-red stained birefringence studies (Fig. 1).

**Figure 1:**
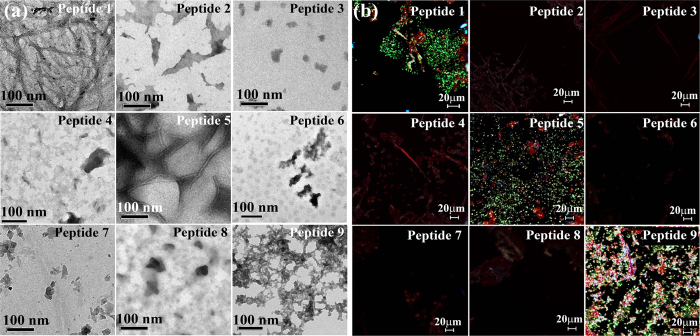
(**a**) TEM and (**b**) Congo red stained birefringence images of the peptides 1–9 respectively. Images were taken after 5 days of incubation in PBS pH 7.4 at 37 °C.

The presence of fibrillar structure under electron microscope (EM) is a characteristic property of amyloid formed by a peptide^32^. Peptide **1** formed clear fibrillar assembly (Fig. 1a) in TEM. But no such fibrillar assembly was observed for peptidomimetics **2**, **3**, **4**, **6**, **7**, and **8**, respectively (Fig. 1a). We observed some fibrillar assembly for peptidomimetic **5** and some amorphous aggregates for peptidomimetic **9** (Fig. 1a). Appearence of green gold birefringence under cross polarized light after staining with Congo red is a property of amyloid and we observed clear green gold birefringence for peptide **1** (Fig. 1b), slight birefringence for peptide **5** and **9** (Fig. 1b), but no such birefringence was observed for breaker peptidomimetics **2**, **3**, **4**, **6**, **7**, and **8** (Fig. 1b)^32^. After 5 days, peptide **1** exhibited β-sheet conformation as evident from the CD and FTIR spectra (Figure S19)^21^. But, such β-sheet conformation was not observed for the peptidomimetics **2**, **3**, **4**, **6**, **7**, and **8** (Figure S19). On the contrary, β-sheet conformations were observed for peptidomimetics **5** and **9** in both CD and FTIR analyses.

The above experimental results suggest that the peptide **1** (NFGAIL) formed amyloid fibrils similar to that of the wild type hIAPP^19^. Out of the peptidomimetics prepared by incorporation of three isomers of aminobenzoic acid, BSBHps containing 2-aminobenzoic acid or 3-aminobenzoic acid (**3**, **4**, **7** and **8**) were found to be non-amyloidogenic. However, BSBHps with 4-aminobenzoic acid (**5** and **9**) exhibited aggregates. This may be accounted for the linear structure of 4-aminobenzoic acid that does not cause any kink formation of the peptide backbone. We further examined the inhibitory efficacy of these designed BSBHps along with the control breaker peptides.

To investigate the efficacy of the BSBHps for the inhibition of aggregation of hIAPP, we initially checked the efficacy of the single breaker element (I26**X**) containing peptidomimetics (**2**, **3**, **4**, and **5**). Later, we continued the investigation with the double breaker elements (**6**, **7**, **8**, and **9**) containing peptidomimetics (G24**X** and I26**X**).

To examine the inhibitory efficacy of the single breaker element (I26**X**) containing BSBHps (**3**, **4** and **5**), we performed various biophysical studies in absence and presence of BSBHps and compared the effect with that of the control **2**. The BSBHps and the control were co-incubated with hIAPP in PBS pH 7.4 at 37 °C up to 7 days and the kinetics of the amyloid formation was monitored using various biophysical tools. To investigate dose dependence, 2-, 5- and 10-fold molar excess of peptidomimetics were incubated with hIAPP.

After 7 days, hIAPP alone exhibited a clear β-sheet conformation both in CD and FTIR (black, Figure S20). But in presence of 10-fold molar excess of BSBHps **3** and **4**, such β-sheet conformations were not observed as evident form CD and FTIR spectra (Figure S20), indicating significant inhibition of aggregation of hIAPP. The control **2** also exhibited significant inhibition. In presence of **5**, β-sheet was observed both in CD and FTIR, indicating no inhibition of aggregation. Similar results were observed when 2-fold and 5-fold molar excess of breakers were co-incubated with hIAPP (Figures S21–S23).

**Figure 2:**
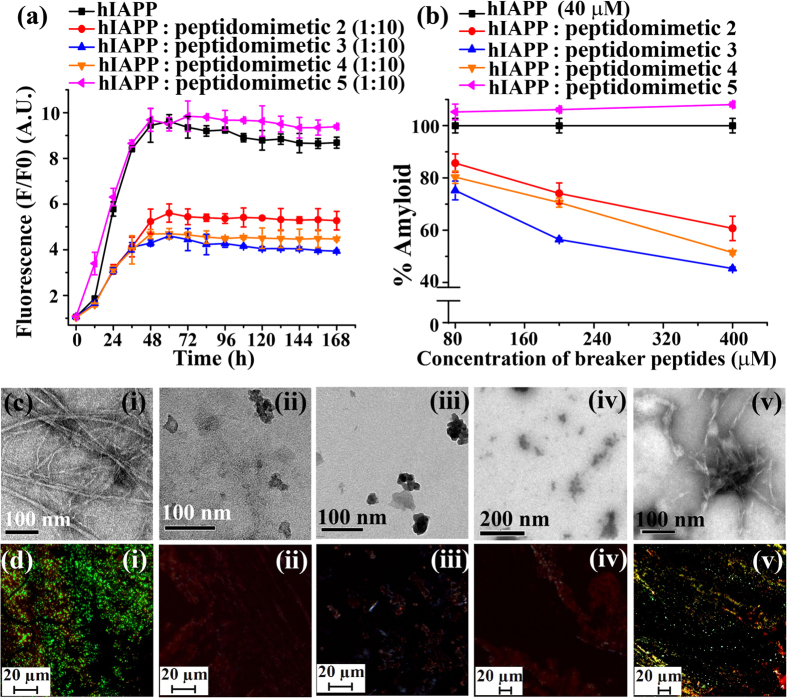
(**a**) Time dependent ThT assay of hIAPP (40 μM) in absence (black) and presence of 10-fold molar excess peptidomimetics, **2** (red), **3** (blue), **4** (orange) and **5** (magenta). (**b**) Dose dependent ThT assay of hIAPP (40 μM) in absence (black) and presence of varied molar excess peptidomimetics, **2** (red), **3** (blue), **4** (orange) and **5** (magenta). (**c**) TEM and (**d**) Congo-red birefringence images of hIAPP (i) alone and in presence of 10-fold molar excess of peptidomimetics, **2** (ii), **3** (iii), **4** (iv) and **5** (v). Images were taken after 7 days of incubation in PBS pH 7.4 at 37 °C.

The hIAPP rapidly formed amyloid aggregates at physiological condition and the kinetics of amyloid formation was monitored by a time dependent Thioflavin T (ThT) assay, which provided quantitative information on the growth of fibril in the sample^32^. The fluorescence intensity was increased when hIAPP was alone in the solution (black, Fig. 2a), while in presence of 10-fold molar excess of **3** (blue, Fig. 2a) the intensity was suppressed up to ~55% (blue, Fig. 2b), indicating the inhibition of amyloid formation. However, in presence of 10-fold molar excess of **4** (orange, Fig. 2a), the inhibition was up to ~50% (orange, Fig. 2b). Similarly, control **2** (red, Fig. 2a) also inhibited the fibril formation considerably, up to ~40% (red, Fig. 2b). But, in presence of **5** (magenta, Fig. 2a) no such inhibition was observed, rather ~5% (magenta, Fig. 2b) enhancement of the intensity was observed. These results indicated that 2-fold molar excess (Figure S24a) of **2**, **3** and **4** were not sufficient to inhibit the amyloid formation of hIAPP. However in presence of 5-fold molar excess (Figure S24b), the inhibition was more pronounced which was further evident when 10-fold molar excess (Fig. 2) was applied.

The hIAPP alone exhibited clear fibrillar structure when viewed under TEM (Fig. 2c(i)), indicating the presence of amyloid. When 10-fold molar excess of **2** (Fig. 2c(ii), **3** (Fig. 2c(iii)) and **4** (Fig. 2c(iv)) were co-incubated with hIAPP, no such fibrillar assembly were observed, indicating significant inhibition of amyloid formation. But when **5** (Fig. 2c(v)) was co-incubated with hIAPP, clear fibril was observed, which indicated the inefficiency of **5** to inhibit the fibrillization of hIAPP. The hIAPP exhibited green gold birefringence under cross polarized light when stained with Congo red (Fig. 2d(i)) indicating amyloid formation by hIAPP. When 10-fold molar excess of **2** (Fig. 2d(ii)), **3** (Fig. 2d(iii)) and **4** (Fig. 2d(iv)) were co-incubated with hIAPP, separately, no such green gold birefringence were observed, indicating inhibition of amyloid formation. However, in presence of **5**, we observed some characteristic green gold birefringence indicating the inefficiency of **5** to inhibit the fibril formation of hIAPP.

The incorporation of single breaker element in BSBHps and their effect for the inhibition of hIAPP aggregation was explained. Next, the impact of double breaker element (G24**X** and I26**X)** incorporated BSBHps for the inhibition of hIAPP were examined in a similar way as described for the single breaker element containing BSBHps. The breaker and the control peptidomimetics were co-incubated with hIAPP in PBS pH 7.4 at 37 °C for 7 days and the kinetics of the amyloid accumulation was monitored using various biophysical tools. After 7 days, hIAPP alone exhibited a clear β-sheet conformation both in CD and FTIR (black, Figure S20 and S25). But in presence of 10-fold molar excess of **7** and **8**, such β-sheet conformations were not observed as evident form CD and FTIR spectra (Figure S25), indicating significant inhibition of aggregation of hIAPP. The corresponding control **6** also exhibited significant inhibition. But in presence of **9** some characteristic β-sheet conformation was observed both in CD and FTIR, indicating no inhibition of aggregation. Similar results were observed when 2-fold and 5-fold molar excess of the peptidomimetics were co-incubated with hIAPP (Figures S26–S28).

**Figure 3:**
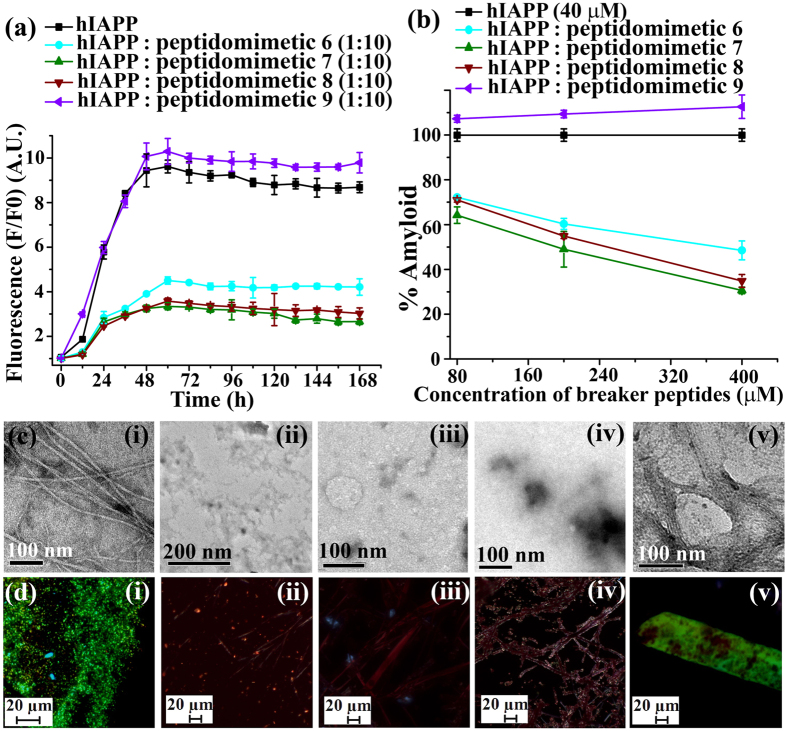
(**a**) Time dependent ThT assay of hIAPP (40 μM) in absence (black) and presence of 10-fold molar excess peptidomimetics, **6** (cyan), **7** (olive), **8** (wine), and **9** (violet). (**b**) Dose dependent ThT assay of hIAPP (40 μM) in absence (black) and presence of varied molar excess peptidomimetics, **6** (cyan), **7** (olive), **8** (wine), and **9** (violet). (**c**) TEM and (**d**) Congo-red birefringence images of hIAPP (i) alone and in presence of 10-fold molar excess of peptidomimetics, **6** (ii), **7** (iii), **8** (iv), and **9** (v). Images were taken after 7 days of incubation in PBS pH 7.4 at 37 °C.

Significant enhancement of fluorescence intensity was observed when hIAPP was alone in the solution (black, Fig. 3a), while in presence of 10-fold molar excess of BSBHp **7** (olive, Fig. 3a) that was suppressed noticeably (up to ~70%, olive, Fig. 3b), indicating inhibition of amyloid formation. The intensity was also significantly suppressed (wine, Fig. 3a; ~65%, wine, Fig. 3b) in presence of 10-fold molar excess of BSBHp **8**. Similarly, control **6** (cyan, Fig. 3a) also inhibited fibril formation reasonably (~52%, cyan, Fig. 3b). But, in presence of **9** (violet, Fig. 3a) no such inhibition was noted, rather ~12% (violet, Fig. 3b) enhancement of fluorescence intensity was observed. We also performed dose dependent ThT assay with 2-fold and 5-fold molar excess of breaker peptidomimetics (Figure S29).

We already observed that hIAPP alone exhibited clear fibrillar morphology in TEM (Fig. 2c (i)), indicating amyloid formation by the peptide. When 10-fold molar excess of **6** (Fig. 3c (ii)), **7** (Fig. 3c(iii)), and **8** (Fig. 3c(iv)) were co-incubated separately with hIAPP, no such fibrillar assembly were observed, indicating inhibition of amyloid formation by them. But when **9** (Fig. 3c(v)) was co-incubated with hIAPP, clear fibril was observed, indicating the inefficacy of **9** to inhibit amyloid formation of hIAPP. Again, hIAPP (Fig. 3d(i)) exhibited green gold birefringence under cross polarized light upon stained with Congo red. When 10-fold molar excess of **6** (Fig. 3d(ii)), **7** (Fig. 3d(iii)), and **8** (Fig. 3d(iv)) were co-incubated with hIAPP, no such green gold birefringence were observed, indicating significant inhibition of amyloid formation. But, the presence of green gold birefringence again supported the inefficiency of **9** to inhibit the amyloid formation of hIAPP.

All the results suggest that the BSBHps **3**, **4**, **7** and **8** (2-Abz and 3-Abz containing analogues) were efficient inhibitors for the amyloid formation of hIAPP. We also observed that incorporation of double breaker elements in BSBHps enhanced their inhibitory efficacy from 55% to 70% (**3** vs **7)**. We did not observe any inhibitory activity of **5** and **9** (4-Abz containing analogues); rather some amyloidogenic properties were noticed. Therefore, the designed peptidomimetic **5** and **9** may not be used as inhibitors.

Since, the amine and carboxylic acid functionalities in 4-Abz (para) is 180° apart from each other and also 4-Abz is a planner molecule, it preferably forms β-sheet structure when present inside the peptide sequence^22^. Whereas, in 2-Abz (ortho) and 3-Abz (meta) the amine and carboxylic acid groups are 60° and 120° apart, respectively. Such orientations do not allow the peptide sequence to form a straight chain or a β-sheet structure rather some distorted structures (helical or turn like structure) evolve^21^. These distorted orientations of the peptidomimetics cannot align with the β-sheet assembly of hIAPP, but disturb its β-sheet assembly and inhibit its aggregation. The para isomer can align with the β-sheet assembly of hIAPP and enhance its aggregation. Therefore, we excluded **5** and **9** (4-Abz containing analogues) from further studies.

We already observed that the BSBHps (**3**, **4**, **7**, and **8**) were efficient inhibitors. Further, we investigated the ability of those molecules to disrupt preformed fibrils of hIAPP *in vitro*. From the ThT assay (black, Fig. 2a), we observed that hIAPP fibrillzation was maximum at ~45–50 h. Therefore, we designed an experiment where the BSBHps were added into the preformed fibrillar assembly at 48 h (2 days). The hIAPP was incubated alone in PBS pH 7.4 at 37 °C for 48 h (2 days) and then varied molar excess (2-, 5-, and 10-fold) of designed peptidomimetics were added to it and fibril disruption was monitored by various biophysical tools.

After 7 (2 + 5) days of incubation, hIAPP exhibited β-sheet conformation both in CD and FTIR (black, Figure S30) analyses. But, in presence of 10-fold molar excess of **2**, **3**, and **4** (Figure S30), such β-sheet conformation disappeared, indicating the significant disruption of amyloid. Similar results were observed when 2-fold and 5-fold molar excess of BSBHps and the control were present with hIAPP (Figures S31–S33).

**Figure 4:**
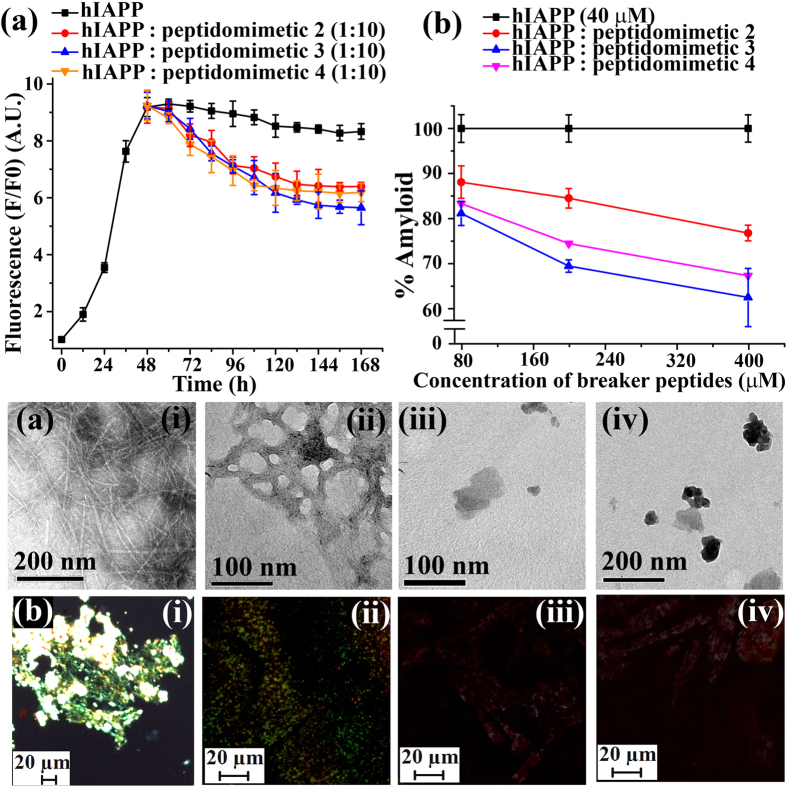
(**a**) Time dependent ThT assay of hIAPP (40 μM) in absence (black) and presence of 10-fold molar excess peptidomimetics, **2** (red), **3** (blue), and **4** (orange). (**b**) Dose dependent ThT assay of hIAPP (40 μM) in absence (black) and presence of varied molar excess peptidomimetics, **2** (red), **3** (blue), and **4** (orange). (**c**) TEM and (**d**) Congo-red birefringence images of hIAPP (i) alone and in presence of 10-fold molar excess of peptidomimetics, **2** (ii), **3** (iii), and **4** (iv). Images were taken after 7 days of incubation in PBS pH 7.4 at 37 °C.

The time dependent ThT fluorescence assay clearly indicated that fluorescence intensity was increased with time when hIAPP was present alone in the solution (black, Fig. 4a) but in presence of 10-fold molar excess of control **2** (red, Fig. 4a) we observed ~23% (red, Fig. 4b) of preformed amyloid disruption. However, in presence of **3** (blue, Fig. 4a,b) ~40% of the preformed amyloid was disrupted. While in presence of **4** (orange, Fig. 4a,b), ~33% of preformed fibrils were disrupted. The dose dependent disruption assays with varied molar excess of BSBHps (Figure S34) was also performed and we observed better result with 10-fold molar excess.

In TEM, hIAPP alone exhibited clear fibrillar structure (Fig. 4c(i)), indicating the presence of amyloid. When 10-fold molar excess of **2** (Fig. 4c(ii)) was present, we noticed some fibrillar assembly, but in presence of **3** (Fig. 4c(iii)) and **4** (Fig. 4c(iv)) in same molar excess we did not observe such fibrillar assembly, indicating significant disruption of preformed amyloid. The hIAPP exhibited green gold birefringence under cross polarized light upon staining with Congo red (Fig. 4d(i)) when incubated alone, indicating presence of amyloid. When 10-fold molar excess of **2** (Fig. 4d(ii)) was present, green gold birefringence still persisted, indicating incomplete disruption. When, **3** (Fig. 4d(iii)) and **4** (Fig. 4d(iv)) were present with hIAPP no such green gold birefringence were observed, indicating significant disruption of amyloid assembly.

The disrupting ability of the double breaker element containing breaker peptides was also performed. After 7 days of incubation, hIAPP showed β-sheet conformation both in CD and FTIR (black, Figure S35) analyses. But, in presence of 10 fold molar excess of **6**, **7**, and **8** (Figure S35), such β-sheet conformation disappeared, indicating the significant disruption of amyloid. Similar results were observed when 2-fold and 5-fold molar excess of BSBHps and the control were present with hIAPP (Figures S36–S38).

**Figure 5:**
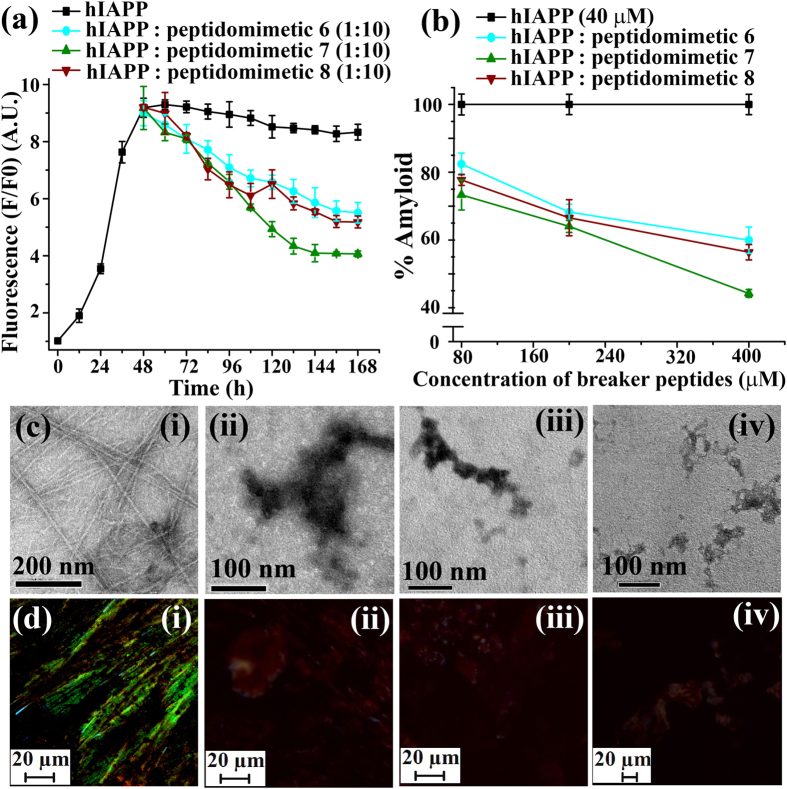
(**a**) Time dependent ThT assay of hIAPP (40 μM) in absence (black) and presence of 10-fold molar excess peptidomimetics, **6** (cyan), **7** (olive) and **8** (wine). (**b**) Dose dependent ThT assay of hIAPP (40 μM) in absence (black) and presence of varied molar excess peptidomimetics, **6** (cyan), **7** (olive) and **8** (wine). (**c**) TEM and (**d**) Congo-red birefringence images of hIAPP (i) alone and in presence of 10-fold molar excess of peptidomimetics, **2** (ii), **3** (iii) and **4** (iv). Images were taken after 7 days of incubation in PBS pH 7.4 at 37 °C.

The time dependent ThT fluorescence assay clearly demonstrated that fluorescence intensity was increased with time when hIAPP was present alone in the solution (black, Fig. 5a) but in presence of 10-fold molar excess of control **6** (red, Fig. 5a) we observed ~40% (cyan, Fig. 5b) of preformed amyloid disruption. In presence of 10-fold molar excess of BSBHp **7** (olive, Fig. 5a) the fluorescence intensity was suppressed significantly; ~57% (olive, Fig. 5b). While in presence of 10-fold molar excess of **8** (wine, Fig. 5a), fluorescence intensity significantly suppressed, ~44% (wine, Fig. 5b), indicating the disruption of preformed fibrillar aggregates. The dose dependent disruption with varied molar excess of BSBHps (Figure S39) was also performed and we observed better result with 10-fold molar excess.

Under TEM, hIAPP alone exhibited clear fibrillar structure (Fig. 5c(i)), indicating presence of amyloid. When 10-fold molar excess of peptidomimetics, **6** (Fig. 5c(ii)), **7** (Fig. 5c(iii)) and **8** (Fig. 5c(iv)) were present with hIAPP, no such fibrillar assembly were observed, indicating significant disruption of preformed amyloid. Again, hIAPP exhibited green gold birefringence under cross polarized light upon staining with Congo red (Fig. 5d(i)) when incubated alone, indicating presence of amyloid. When 10-fold molar excess of **6** (Fig. 5d(ii), **7** (Fig. 5d(iii) and **8** (Fig. 5d(iv) were present with hIAPP no such green gold birefringence were observed, indicating significant disruption of amyloid assembly.

From the systematic studies on the preformed fibril disrupting capabilities of the BSBHps, we observed that BSBHps **3**, **4**, **7** and **8** were efficient to disrupt the preformed fibrils of hIAPP. We also observed that the incorporation of double breaker elements in BSBHps enhanced its fibril disrupting capability from 40% to 57% (**3 **vs **7)**.

Peptidomimetics containing double breaker elements (insertions at two positions, G24X and I26X) were more efficient in inhibiting the hIAPP aggregation as well as disruption of preformed fibrillar aggregates as observed by time dependent ThT assay.

**Figure 6:**
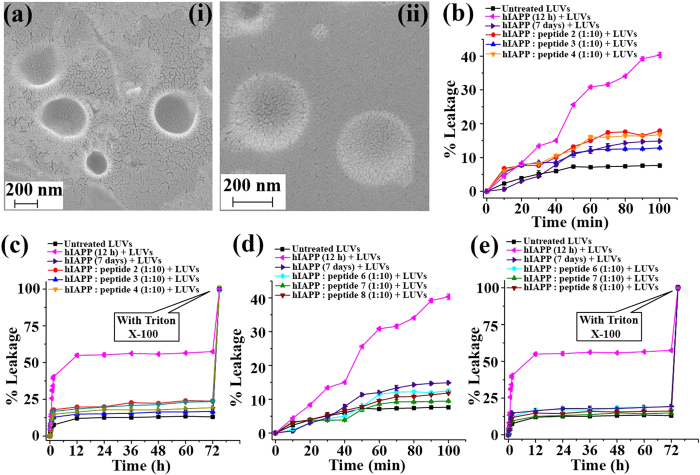
(**a**) FE-SEM images (i & ii) of the large unilamellar vesicles (LUVs) at concentration of 1 mM in HEPES buffer (50 mM). Scale bar is indicated as 200 nm. Images were taken after imidiate preparation of the vesicles. Carboxyfluorescein dye emission showing the effect of hIAPP on LUVs with time and % of dye leakage. (**b**,**d**) release of dye from LUVs in absence and presence of different samples from 0 min to 100 min. (**c**,**e**) release of dye from LUVs in absence and presence of different samples from 0 min to 72 h.

The hIAPP soluble oligomers are known as more toxic than the matured amyloid, since they can damage the cell by forming pores on the cell membrane^9^,^10^,^33^. We have performed a dye leakage assay using carboxyfluorescein entrapped large unilamellar vesicles (LUVs, Fig. 6a(i & ii))^34^,^35^,^36^ and found that BSBHps efficiently re-dissolved pre-formed hIAPP fibrils into non-toxic species (Fig. 6b–e). To perform the vesicle leakage study, we have prepared 9 sets of different samples including untreated LUVs (without any peptide) as control for the experiment. All the peptidomimetics were added to hIAPP solution after 48 h of incubation and kept for total of 7 days (168 h = 48 h + 120 h) of incubation. After that different peptide solutions were added separately to the LUVs and leakage studies were performed. The different type of samples that were prepared is described below;

Sample 1 Untreated LUVs

Sample 2- LUVs + hIAPP (incubated for 12 h),

Sample 3- LUVs + hIAPP (incubated for 7 days)

Sample 4- LUVs + hIAPP : peptidomimetic **2** (1:10)

Sample 5- LUVs + hIAPP : peptidomimetic **3** (1:10)

Sample 6- LUVs + hIAPP : peptidomimetic **4** (1:10)

Sample 7- LUVs + hIAPP : peptidomimetic **6** (1:10)

Sample 8- LUVs + hIAPP : peptidomimetic **7** (1:10)

Sample 9- LUVs + hIAPP : peptidomimetic **8** (1:10)

The peptide and the lipid were present in 1:20 molar ratios during leakage analysis. At the end of the experiment 10 μL of Triton X-100 was added to obtain complete dye release from the vesicle and the final fluorescence was measured. In addition to those, untreated LUVs (natural dye leakage) were studied and used as control. % dye leakage was calculated as^35^,


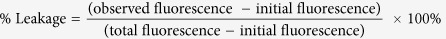


We observed rapid increment of dye leakage of LUVs treated with hIAPP (sample 2) from 0 to 100 min but such increment was not observed after 24 h while the natural leakage of LUVs was saturated after 50 min. The spectra showing % of dye leakage by untreated LUVs (black), sample 2 (magenta), sample 3 (purple), sample 4 (red), sample 5 (blue), sample 6 (orange), sample 7 (cyan), sample 8 (olive) and sample 9 (wine). The 100% dye release was obtained by treating the LUVs with triton X-100. The composition of samples 1–9 was mentioned above.

It was evident from the results of the dye leakage tests from LUVs that the oligomers formed after 12 hours of incubation of hIAPP were more toxic than the mature fibrils obtained after 7 days of incubation. On the other hand, fibril disrupted by the control peptide and the designed BSBHps could not form pore on the artificial membranes or LUVs significantly, as the increment of fluorescence in corresponding samples were as low as the untreated LUVs (sample 1, black). This result was indicative of non-toxic nature of the disrupted preformed fibrils of hIAPP by the BSBHps.

Further, to test the stability of these peptidommetics in biological system we performed *in vitro* stability study in presence of human serum^37^,^38^ which contained sufficient concentration of proteolytic enzymes. We selected BSBHPs **3** and **7**, as from the results of all the experiments described above it was clear that BSBHps **3** (one breaker element containing) and **7** (two breaker elements containing) were the best among all the synthesized peptidomimetics. We compared the stability of **3** and **7** with their native peptide analogue (**1**) using time dependent HPLC & Mass spectrometry studies. In presence of the proteolytic enzyme the native peptide (**1**) started degrading immediately and after 1 h we did not observe any trace of it indicating complete degradation (Figure S40–S41). The BSBHp **3** was found to be relatively more stable in presence of proteolytic enzyme; some degradation was observed at 25 h (Figure S42–S43). On the other hand, BSBHp **7**, which bears two breaker Abz units, was found to be more stable than **3**. We did not observe any trace of degradation of 7, rather complete retention of the peak in HPLC of the pure compound was noted till 25 h (Figure S44–S45). Above results indicate indeed insertion of Abz unit increases the stability of the peptide.

**Figure 7:**
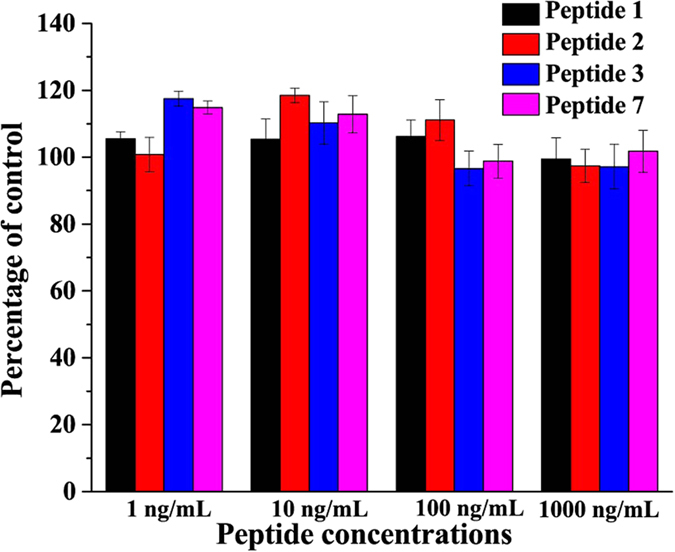
Effects of the peptides **1**, **2**, **3** and **7** on human embryonic kidney (HEK) cells in culture and cell viability assay using MTT reduction after 36 and 72 hours treatments.

To check whether the BSBHps are toxic to the mammalian cells, we have tested their effect on human embryonic kidney (HEK) cells in culture and determined the cell viability using MTT assay after 36 and 72 hours treatments. The results showed that up to 1 μM concentrations of peptides, which is approximately million fold higher than the physiological concentration of Amylin (at the range of pmol/L)^39^, did not alter the cell viability (Fig. 7).

## Discussion

We previously reported that the anthranilic acid containing conformationally restricted β-sheet breaker α/β-hybrid peptides are potent inhibitors of the aggregation of Alzheimer’s β-amyloid^23^. Since, conformationally restricted peptides^17^,^18^,^40^,^41^,^40^ showed excellent efficacy for the inhibition of amyloid formation in T2DM, some of those peptides were used for clinical trial and used as potent drug against T2DM^40^,^41^,^42^. We have described here a novel class of β-sheet breaker hybrid peptidomimetics (BSBHps) as potent inhibitors of amyloid formation of wild type hIAPP and its reversion.

We prepared various peptidomimetics incorporating three different isomers of amino benzoic acids and from the experimetal results it was clear that the ortho (2-Abz) and meta (3-Abz) isomer containing BSBHps were non-amyloidogenic at physiological condition (pH 7.4 and 37 °C), whereas the para (4-Abz) isomer containing BSBHps showed some amorphous or amyloid aggregates. The experimental results also supported that ortho (2-Abz) and meta (3-Abz) isomer containing BSBHps were potent inhibitors against amyloid formation of hIAPP, but the para (4-Abz) isomer did not act as inhibitor rather it enhenced the aggregation process. Therefore, we excluded the 4-Abz containing BSBHps for further studies against amylin aggregation.

Further, we demonstrated two types of BSBHps, one containing single breaker element and another containing double breaker elements. We observed that the double breaker element containing BSBHps were more efficent than the single breaker element containing BSBHps. We have also demonstrated that these BSBHps efficienlty disrupt the preformed fibrillar aggregate of hIAPP into non-toxic species.

Moreover, we demonstrated that the BSBHps (**3** & **7**) were more stable than their native peptide analogue (**1**) in presence of proteolytic enzymes. The enhencement of the stability of the BSBHps were presumably due to the presence of non-proteinogenic amino acid, Abz, in the peptide sequence, which was unlikely to get recognised by the proteases. We also showed the non-toxic nature of the BSBHps on mammalian cells in culture.

Since, β and γ-amino acids are non-proteinnogenic in nature and the BSBHps containing these amino acids showed better inhibitory activity than the tetra substituted α-amino acid (Aib) containing breaker peptides, these results can be used for designing Amylin agonists with better pharmacodynamic as well as pharmacokinetic profile that can be used for drug design against T2DM. Further investigations in this direction will be communicated in the due course of time. Furthermore, similar strategies may be used for drug design against other amyloidoses and understanding amyloidogenesis.

## Methods

### Peptide synthesis

All the peptides described here were synthesized by standard Fmoc/^t^Bu solid phase peptide synthesis method on MBHA-Rink amide resin (loading 0.7 mmol/g). For each amino acid attachment, 2 equiv of Fmoc amino acids, 2.5 equiv of coupling reagent (BOP), and 5 equiv of base (DIPEA) were used and for incomplete reaction, coupling cycles were repeated, followed by capping with acetic anhydride (2 equiv) and N-methyl imidazole (3 equiv). Fmoc deprotection was performed with 20% piperidine in DMF. The final peptide was cleaved from the resin using a cleavage cocktail (90% TFA, 5% DCM and 5% H_2_O) for 5 h. The crude peptide was precipitated by cold diethyl ether followed by centrifugation to get the crude solid peptide.

### Liquid chromatography and mass spectrometry

Crude peptides were purified by RP-HPLC (Waters 600E) using a semi-preparative C18-μ Bondapak column at a flow rate of 5** **mL/ min. Binary solvent system were used, solvent A (0.1% TFA in H_2_O) and solvent B (0.1% TFA in CH_3_CN). A Waters 2489 UV detector was used with dual detection at 214 and 254 nm. A total run time of 20 min was used and gradient used for purification was 5–100% CH_3_CN for 18 min followed by 100% CH_3_CN till 20 min. Purity of the peptides were confirmed using Waters 600E analytical HPLC system using Waters C8 analytical column at a flow rate of 1 ml/min, linear gradient of 5–100% CH_3_CN over 18 minutes in a total run time of 20 min. The purified peptides were characterized by mass spectrometry on Agilent-Q-TOF 6500 instrument, in ESI positive mode, equipped with Mass hunter work station software.

### hIAPP Sample preparation

3.1 mg of hIAPP_(1-37)_ was dissolved in 50 μL of HFIP to obtain disaggregated hIAPP and HFIP was evaporated using nitrogen gas^43^. This process was repeated twice. Then, 5 mL of Milli Q water was added to the disaggregated material and divided the whole solution into 25 equal portions with 200 μL each. All 25 portions are lyophilized followed by addition of 800 μL of PBS (50 mM, pH 7.4) to each portion to obtain a final hIAPP concentration of 40 μM.

### Thioflavin T Fluorescence Assay

50 μM Thioflavin T (ThT) in PBS (50 mM, pH 7.4) was prepared^32^. All the synthesized peptides and hIAPP (alone and in some cases mixed with breaker peptides) were dissolved in PBS (50 mM, pH 7.4) to obtain a desired stock solution (40 μM in the case of hIAPP) and incubated at 37 °C over a water bath. To perform the fluorescence study, 40 μL of sample was taken out from the stock solution and was mixed with 200 μL of ThT solution (50 μM); final volume was made up to 400 μL with PBS. For ThT fluorescence assay, emission was measured at 490 nm and excitation at 435 nm, using a slit of 3 nm on a Fluoromax-4, Horiba instrument. The text files were taken from the instrument and graphs were plotted using OriginPro 8 software. For each data point 3 different sets of replica were scanned separately and average was taken with observed standard deviation.

### Transmission Electron Microscopy (TEM)

10 μL aliquot from the stock peptide solution was added over the dark side of carbon coated copper grid and followed by addition of 2% uranyl acetate solution (10 μL) and was allowed to float for 1 min^32^. The excess solution was removed using blotting paper. The sample was dried at room temperature and was kept in desiccator before taking TEM analysis on JEOL (Model: JEM 2100) instrument at 200 kV.

### Congo-Red Stained Birefringence

A 10 μL aliquot of the desired peptide solution was placed over a glass slide followed by 20 μL of the saturated Congo red solution (in 80% aqueous ethanol)^32^. The excess solution was removed using a blotting paper; the sample was dried at room temperature and analysed under a Leica ICC50 HD polarizable microscope.

### Circular dichroism (CD)

To perform the CD study, 400 μL of the sample was taken in a cuvette and measured ellipticity with bandwidth of 1 mm^20^. Three measurements were accumulated. Spectra were recorded from 190 nm to 260 nm on a JASCO J-815 instrument. Observed ellipticity (mDeg) was converted to mean residue molar ellipticity using the following equation:


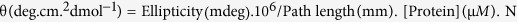


### Fourier transformation infra-red (FT-IR)

An aliquot of 20 μL was taken out from the stock peptide solution, mixed with KBr, and a pellet was prepared^32^. The spectrum for each sample was recorded immediately after sample preparation. The background scan was subtracted from the sample scans and text files were plotted using OriginPro 8 software.

### Large Unilamellar Vesicles (LUVs) Preparation and Carboxyfluorescein Entrapment

The LUVs were prepared using three different lipids^34^,^35^, DPPC, Cholesterol and GM1 with 68:30:2 molar ratios in 50 mM HEPES buffer of pH 7.4. Prior to the LUVs preparation, all the lipids were taken in clean glass vessel and solubilize to make 2 mM stock solution in chloroform and methanol (2:1) and the solvents were evaporated under vacuum. The lipid films were hydrated with 500 μL of carboxyfluorescein solution (200 μM) in 50 mM HEPES buffer of pH 7.4. Then, the solution was vortexed vigorously for 30 min to emulsify the lipid mixtures. Further, the glass vessel was dipped into the liquid nitrogen for instant cooling and after 5 min the frozen solution was dipped into water bath at 50–60 °C for thawing^36^. This step was repeated five times. The excess dye was removed by ultracentrifugation at 20000 rpm and the supernatant dye solution was discarded and the lipid pellet was re-hydrated with 50 mM HEPES buffer. This step was repeated twice more and the final lipid pellet was collected followed by addition of 500 μL of HEPES buffer and vortexed to obtain homogenous suspension of 2 mM of dye loaded LUVs. The leakage study was performed on a Fluoromax-4, Horiba instrument. The formation of LUVs was confirmed by electron microscopy.

### Field Emission Scanning Electron Microscope (FESEM)

10 μL lipid vesicle solution was added over the cleaned glass slide covered with aluminium foil and was allowed to dry at room temperature and kept in desiccator before taking FESEM analysis on Ziess, Sigma VP instrument.

### Stability Analysis of the BSBHps in presence of proteolytic enzymes

1 mL of the RPMI (Roswell Park Memorial Institute) 1640 media supplemented with 10% human serum (v/v) obtained from Thermo Fisher Scientific was taken in three different 2 mL eppendorf tube and incubated them at 37 °C for 15 min^38^. Then, 10 μL of the peptidomimetics (**1**, **3** & **7**) in DMSO (5 mg/mL) were separately added to the RPMI media and incubated them at 37 °C. At different time interval 100 μL of the aliquot was taken out from the three different eppendorf tubes and added to 200 μL ethanol solution (96% aqueous ethanol) for precipitation of the serum protein. The precipitate was cooled (4 °C) for 10 min and then centrifuged at 18,000 rpm for 2 min and the pellet was discarded. The supernatant was collected and analyzed by RP-HPLC & Mass spectrometry. A linear gradient was used in the HPLC, from 5–100% CH_3_CN for 18 min followed by 100% CH_3_CN till 20 min with a flow rate of 4 mL/min using a semi-preparative C18-μ Bondapak column. Binary solvent system were used, solvent A (0.1% TFA in H_2_O) and solvent B (0.1% TFA in CH_3_CN).

### Cell viability/proliferation assay

Human embryonic kidney (HEK) cells were grown in Dulbecco’s Modified Eagle’s medium (DMEM)-F12 + GLUTAMAX (Gibco) medium containing 10% fetal calf serum (FCS; Biowhitaker), 100 μg/ml penicillin and 100 μg/ml streptomycin (Gibco). Cells were maintained at 37 °C under 95% air and 5% CO_2_. For 3-(4,5-dimethylthiazol-2-yl)-2,5-diphenyltetrazolium bromide (MTT; Sigma) based cell viability assay, we seeded 1000-5000 cells per well in 96 well plates and incubated with different concentrations of peptides at 37 °C in a CO_2_ incubator. After 36 or 72 hours, the cell density was assessed using a plate reader (absorbance at 570 nm) after the addition of MTT and DMSO.

## Additional Information

**How to cite this article**: Paul, A. *et al*. Disaggregation of Amylin Aggregate by Novel Conformationally Restricted Aminobenzoic Acid containing α/β and α/γ Hybrid Peptidomimetics. *Sci. Rep.* **7**, 40095; doi: 10.1038/srep40095 (2017).

**Publisher's note:** Springer Nature remains neutral with regard to jurisdictional claims in published maps and institutional affiliations.

## Supplementary Material

Supporting Information

## Figures and Tables

**Table 1:** Sequences of synthesized peptides for the present study.

Peptide no.	Peptide sequence	Molecular mass [M + H]^+^ (expected/observed)	Function
**1**	NFGAIL	633.3724/633.3748	Aggregating/control
**2**	NFGAX_1_L	605.3511/605.3627	inhibitor/control
**3**	NFGAX_2_L	639.3255/639.3310	inhibitor
**4**	NFGAX_3_L	639.3255/639.3315	inhibitor
**5**	NFGAX_4_L	639.3555/639.3335	inhibitor
**6**	NFX_1_AX_1_L	633.3724/633.3749	inhibitor/control
**7**	NFX_2_AX_2_L	701.3411/701.3414	inhibitor
**8**	NFX_3_AX_3_L	701.3411/701.3410	inhibitor
**9**	NFX_4_AX_4_L	701.3411/701.3419	inhibitor

N.B. Standard amino acids are represented by one letter code, X_1_ = α-aminoisobutyric acid (Aib), X_2_ = 2-aminobenzoic acid (2-Abz), X_3_ = 3-aminobenzoic acid (3-Abz) and X_4_ = 4-aminobenzoic acid (4-Abz).

## References

[b1] Knowles T. P. J., Vendruscolo M. & Dobson C. M. The amyloid state and its association with protein misfolding diseases. Nat. Rev. Mol. Cell Biol. 15, 384–396 (2014). 24854788 10.1038/nrm3810

[b2] Aguzzi A. & O’Conno T. Protein aggregation diseases: pathogenicity and therapeutic perspectives. Nat. Rev. Drug Discovery, 9, 237–248 (2010). 20190788 10.1038/nrd3050

[b3] Squires A. M. . X-ray scattering study of the effect of hydration on the cross-beta structure of amyloid fibrils. J. Am. Chem. Soc. 13, 11738–11739 (2006). 10.1021/ja063751v 16953596

[b4] Lewandowski J. R. . Structural Complexity of a Composite Amyloid Fibril. J. Am. Chem. Soc. 133, 14686–14698 (2011). 21766841 10.1021/ja203736z PMC3190136

[b5] Lansbury P. T. Jr. In pursuit of the molecular structure of amyloid plaque: new technology provides unexpected and critical information. Biochemistry, 31, 6865–6870 (1992). 1637821 10.1021/bi00145a001

[b6] Pilkington E. H. . Pancreatic β-Cell Membrane Fluidity and Toxicity Induced by Human Islet Amyloid Polypeptide Species. Sci Rep. 6, 21274 (2016). 26880502 10.1038/srep21274 PMC4754679

[b7] Westermark P., Andersson A. & Westermark G. T. Islet amyloid polypeptide, islet amyloid, and diabetes mellitus. Physiol. Rev. 91, 795–826 (2011). 21742788 10.1152/physrev.00042.2009

[b8] Kapurniotu A. Amyloidogenicity and cytotoxicity of islet amyloid polypeptide. Biopolymers, 60, 438–459 (2001). 12209476 10.1002/1097-0282(2001)60:6<438::AID-BIP10182>3.0.CO;2-A

[b9] Brender J. R., Salamekh S. & Ramamoorthy A. Membrane disruption and early events in the aggregation of the diabetes related peptide IAPP from a molecular perspective. Acc. Chem. Res. 45, 454–462 (2012). 21942864 10.1021/ar200189b PMC3272313

[b10] Anguiano M., Nowak R. J. & Lansbury P. T. Jr. Protofibrillar Islet Amyloid Polypeptide Permeabilizes Synthetic Vesicles by a Pore-like Mechanism that May Be Relevant to Type II Diabetes. Biochemistry, 41, 11338–11343 (2002). 12234175 10.1021/bi020314u

[b11] Nossol M. T. . Inhibition of hIAPP amyloid-fibril formation and apoptotic cell death by a designed hIAPP amyloid-core-containing hexapeptide. Chem. Biol. 12, 797–809 (2005). 16039527 10.1016/j.chembiol.2005.05.010

[b12] Hard T. & Lendel C. Inhibition of amyloid formation. J. Mol. Biol. 421, 441–465 (2012). 22244855 10.1016/j.jmb.2011.12.062

[b13] Rajasekhar K., Chakrabarti M. & Govindaraju T. Function and toxicity of amyloid beta and recent therapeutic interventions targeting amyloid beta in Alzheimer’s disease. Chem. Commun. 51, 13434–13450 (2015). 10.1039/c5cc05264e 26247608

[b14] Tenidis K. . Identification of a penta- and hexapeptide of islet amyloid polypeptide (IAPP) with amyloidogenic and cytotoxic properties. J. Mol. Biol. 295, 1055–1071 (2000). 10656810 10.1006/jmbi.1999.3422

[b15] Westermark P. . Islet amyloid polypeptide: pinpointing amino acid residues linked to amyloid fibril formation. Proc. Natl. Acad. Sci. USA 87, 5036–5040 (1990). 2195544 10.1073/pnas.87.13.5036 PMC54256

[b16] Abedini A., Meng F. & Raleigh D. P. A Single-Point Mutation Converts the Highly Amyloidogenic Human Islet Amyloid Polypeptide into a Potent Fibrillization Inhibitor. J. Am. Chem. Soc. 129, 11300–11301 (2007). 17722920 10.1021/ja072157y

[b17] Gilead S. & Gazit E. Inhibition of Amyloid Fibril Formation by Peptide Analogues Modified with α-Aminoisobutyric Acid. Angew. Chem. Int. Ed. 43, 4041–4044 (2004). 10.1002/anie.200353565 15300690

[b18] Mishra A. . Conformationally restricted short peptides inhibit human islet amyloid polypeptide (hIAPP) fibrillization. Chem Commun. 49, 2688–2690 (2013). 10.1039/c3cc38982k PMC3684849 23435449

[b19] Kapurniotu A., Schmauder A. & Tenidis K. Structure-based design and study of non-amyloidogenic, double N-methylated IAPP amyloid core sequences as inhibitors of IAPP amyloid formation and cytotoxicity. J. Mol. Biol. 315, 339–350 (2002). 11786016 10.1006/jmbi.2001.5244

[b20] Nadimpally K. C., Paul A. & Mandal B. Reversal of Aggregation Using β-Breaker Dipeptide Containing Peptides: Application to Aβ_(1–40)_ Self-Assembly and Its Inhibition. ACS Chem. Neurosci. 5, 400–408 (2014). 24661180 10.1021/cn500064z PMC4030796

[b21] Pravakaran P. . Sequence-Specific Unusual (1→2)-Type Helical Turns in α/β-Hybrid Peptides. J. Am. Chem. Soc. 130, 17743–17754 (2008). 19061328 10.1021/ja804297f

[b22] Maity S. . Conformational Heterogeneity, Self-Assembly, and Gas Adsorption Studies of Isomeric Hybrid Peptides. Cryst. Growth. Des. 12, 422–428 (2012).

[b23] Paul A. . Inhibition of Alzheimer’s amyloid-β peptide aggregation and its disruption by a conformationally restricted α/β hybrid peptide. Chem. Commun. 51, 2245–2248 (2015). 10.1039/c4cc09063b 25514992

[b24] Haynes S. W. . Assembly of Asperlicin Peptidyl Alkaloids from Anthranilate and Tryptophan: A Two-Enzyme Pathway Generates Heptacyclic Scaffold Complexity in Asperlicin E. J. Am. Chem. Soc. 134, 17444–17447 (2012). 23030663 10.1021/ja308371z PMC3500603

[b25] Oza V. B. . Synthesis and evaluation of anthranilic acid-based transthyretin amyloid fibril inhibitors. Bioorg. Med. Chem. Lett. 9, 1–6 (1999). 9990446 10.1016/s0960-894x(98)00696-9

[b26] Simons L. J. . The synthesis and structure–activity relationship of substituted N-phenyl anthranilic acid analogs as amyloid aggregation inhibitors. Bioorg. Med. Chem. Lett. 19, 654–657 (2009). 19121939 10.1016/j.bmcl.2008.12.049

[b27] Cheng R. P., Gellman S. H. & DeGrado W. F. β-Peptides: From Structure to Function. Chem. Rev. 101, 3219–3232 (2001). 11710070 10.1021/cr000045i

[b28] Shen Y. . Mass Spectrometry Analysis of Proteome-Wide Proteolytic Post-Translational Degradation of Proteins. Anal. Chem. 80, 5819–5828 (2008). 18578501 10.1021/ac800077w PMC2716136

[b29] Formaggio F. . Disruption of the β-sheet structure of a protected pentapeptide, related to the β-amyloid sequence 17–21, induced by a single, helicogenic Cα-tetrasubstituted α-amino acid. J. Peptide Sci. 9, 461–466 (2003). 12916643 10.1002/psc.503

[b30] Moretto V. . Comparison of the Effect of Five Guest Residues on the β-Sheet Conformation of Host (L-Val)_n_ Oligopeptides. Macromolecules, 22, 2939–2944 (1989).

[b31] Yan L. M. . Design of a mimic of nonamyloidogenic and bioactive human islet amyloid polypeptide (IAPP) as nanomolar affinity inhibitor of IAPP cytotoxic fibrillogenesis. Proc. Natl. Acad. Sci. USA 103, 2046–2051 (2006). 16467158 10.1073/pnas.0507471103 PMC1413694

[b32] Nilsson M. R. Techniques to study amyloid fibril formation *in vitro*. Methods, 34, 151–160 (2004). 15283924 10.1016/j.ymeth.2004.03.012

[b33] Cao P. . Islet amyloid polypeptide toxicity and membrane interactions. Proc. Natl. Acad. Sci. USA 110, 19279–19284 (2013). 24218607 10.1073/pnas.1305517110 PMC3845181

[b34] Williams T. L., Day I. J. & Serpell L. C. The effect of Alzheimer’s Aβ aggregation state on the permeation of biomimetic lipid vesicles. Langmuir, 26, 17260–17268 (2010). 20923185 10.1021/la101581g

[b35] McLaurin J. & Chakrabartty A. Membrane disruption by Alzheimer beta-amyloid peptides mediated through specific binding to either phospholipids or gangliosides. Implications for neurotoxicity. J. Biol. Chem. 271, 26482–26489 (1996). 8900116 10.1074/jbc.271.43.26482

[b36] Traikia M. . Formation of unilamellar vesicles by repetitive freeze-thaw cycles: characterization by electron microscopy and 31P-nuclear magnetic resonance. Eur. Biophys. J. 29, 184–195 (2000). 10968210 10.1007/s002490000077

[b37] Frackenpohl J. . The outstanding biological stability of beta-and gamma-peptides toward proteolytic enzymes: an *in vitro* investigation with fifteen peptidases. Chembiochem. 2, 445–455 (2001). 11828476 10.1002/1439-7633(20010601)2:6<445::aid-cbic445>3.3.co;2-i

[b38] Jenssen H. & Aspmo S. I. Serum stability of peptides. Methods Mol Biol. 494, 177–186 (2008). 18726574 10.1007/978-1-59745-419-3_10

[b39] Percy A. J. . Development of sensitive immunoassays to detect amylin and amylin-like peptides in unextracted plasma. Clin Chem. 42, 576–585 (1996). 8605675

[b40] Jones M. C. Therapies for diabetes: pramlintide and exenatide. Am. Fam. Physician. 75, 1831–1835 (2007). 17619527

[b41] Ryan G. J., Jobe L. J. & Martin R. Pramlintide in the treatment of type 1 and type 2 diabetes mellitus. Clin. Ther. 27, 1500–1512 (2005). 16330288 10.1016/j.clinthera.2005.10.009

[b42] Hollander P. . Effect of Pramlintide on Weight in Overweight and Obese Insulin-Treated Type 2 Diabetes Patients. Obesity 12, 661–668 (2004). 10.1038/oby.2004.76 15090634

[b43] Highama C. E. . Preparation of synthetic human islet amyloid polypeptide (IAPP) in a stable conformation to enable study of conversion to amyloid-like fibrils. FEBS Letters 470, 55–60 (2000). 10722845 10.1016/s0014-5793(00)01287-4

