# Supplementary material for: Disaggregation of Amylin Aggregate by Novel Conformationally Restricted Aminobenzoic Acid containing α/β and α/γ Hybrid Peptidomimetics
Source: Sci Rep. 2017 Jan 5;7:40095. doi: 10.1038/srep40095 (PMC5214534; doi:10.1038/srep40095)
Supplement: Supporting Information [file srep40095-s1.pdf]

## Supporting Information

# Disaggregation of Amylin Aggregate by Novel Conformationally Restricted Aminobenzoic Acid containing α/β and α/γ Hybrid Peptidomimetics

**Ashim Paul[1], Sourav Kalita[1], Sujan Kalita[1], Piruthivi Sukumar[2&3], and Bhubaneswar Mandal[1,*]**

[1]Laboratory of Peptide and Amyloid Research, Department of Chemistry, Indian Institute of Technology Guwahati, Assam- 781039, India. Email: bmandal@iitg.ernet.in

[2]Department of Biosciences and Bioengineering, Indian Institute of Technology Guwahati, Assam-781039, India.

3Leeds Institute of Cardiovascular and Metabolic Medicine, LIGHT Laboratories, University of Leeds, Leeds LS2 9JT, UK.

# Characterization data of peptides 1 to 9:

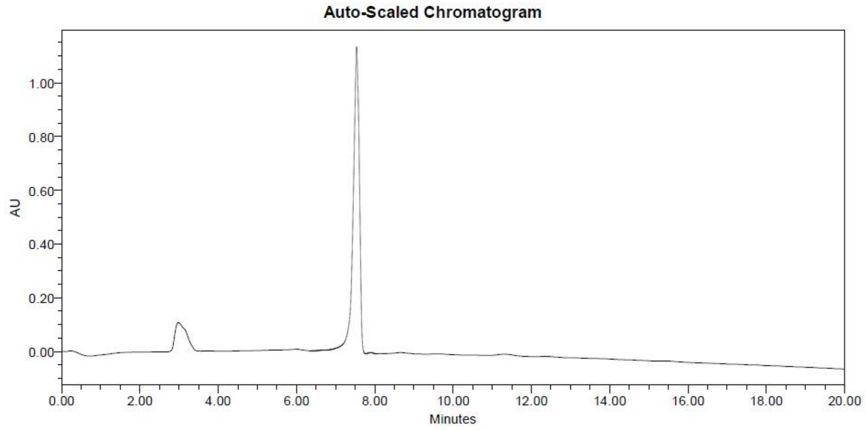


**Figure S1 :** HPLC profile of the purified peptide **1**.

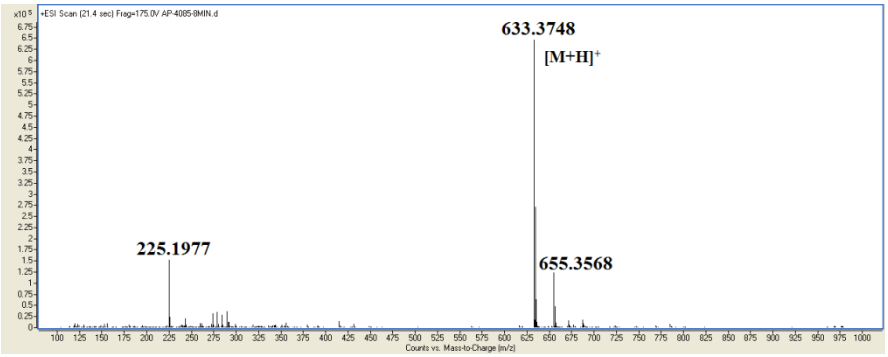


**Figure S2.** Mass spectrum of peptide **1**. Calculated mass for $C_{30}H_{49}N_8O_7$ is 633.3724 $[M+H]^+$, observed 633.3748 $[M+H]^+$ and calculated mass for $C_{30}H_{48}N8O_7Na$ is 655.3544 $[M+Na]^+$, observed 655.3568 $[M+Na]^+$.

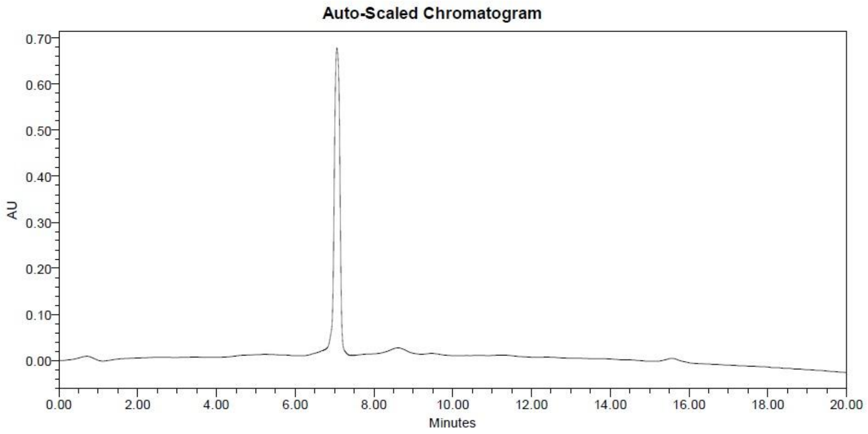


**Figure S3.** HPLC profile of the purified peptidomimetic **2**.

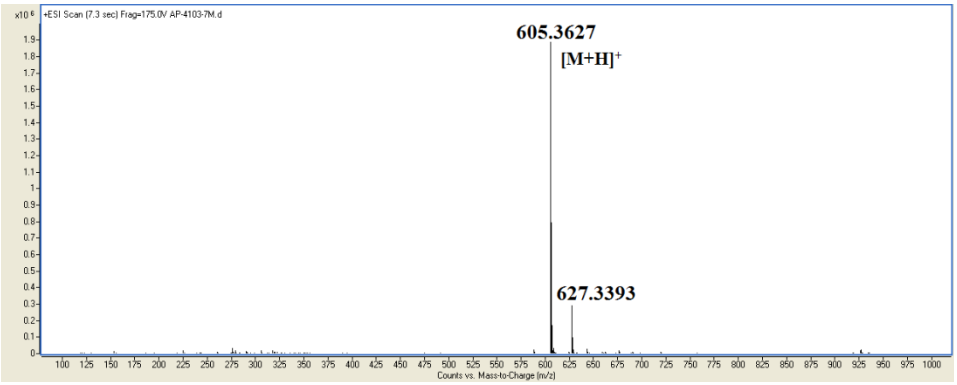


**Figure S4.** Mass spectrum of peptidomimetic **2**. Calculated mass for $C_{28}H_{45}N_8O_7$ is 605.3411 $[M+H]^+$, observed 605.3627 $[M+H]^+$ and calculated mass for $C_{28}H_{44}N_8O_7Na$ is 627.3231 $[M+Na]^+$, observed 627.3393 $[M+Na]^+$.

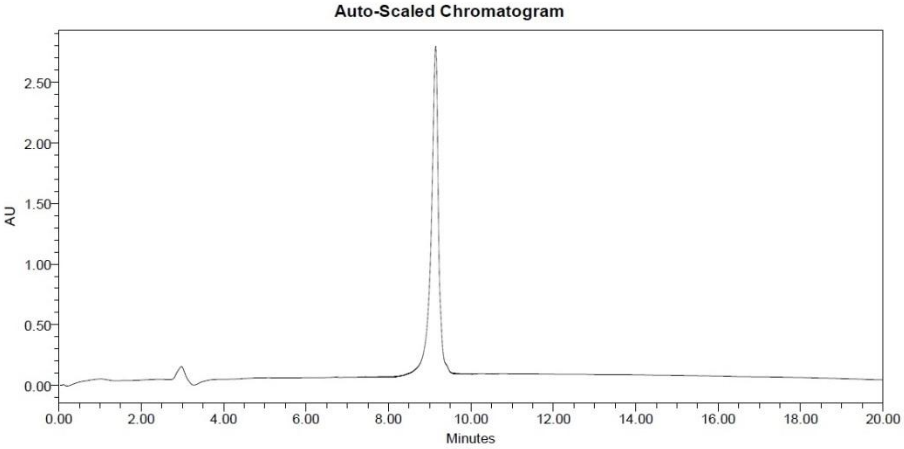


**Figure S5.** HPLC profile of the purified peptidomimetic **3**.

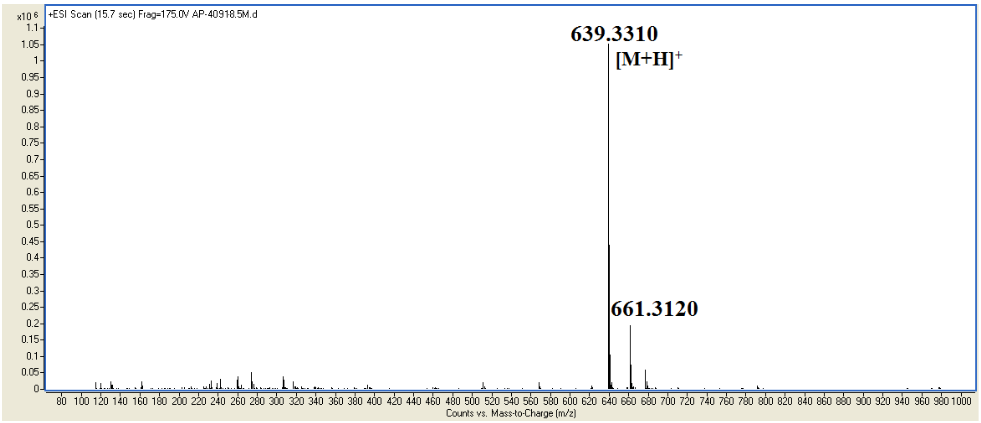


**Figure S6.** Mass spectrum of peptidomimetic **3**. Calculated mass for $C_{31}H_{43}N_8O_7$ is 639.3255 $[M+H]^+$, observed 639.3310 $[M+H]^+$ and calculated mass for $C_{31}H_{42}N_8O_7Na$ is 661.3074 [M+Na]+, observed 661.3120 $[M+Na]^+$.

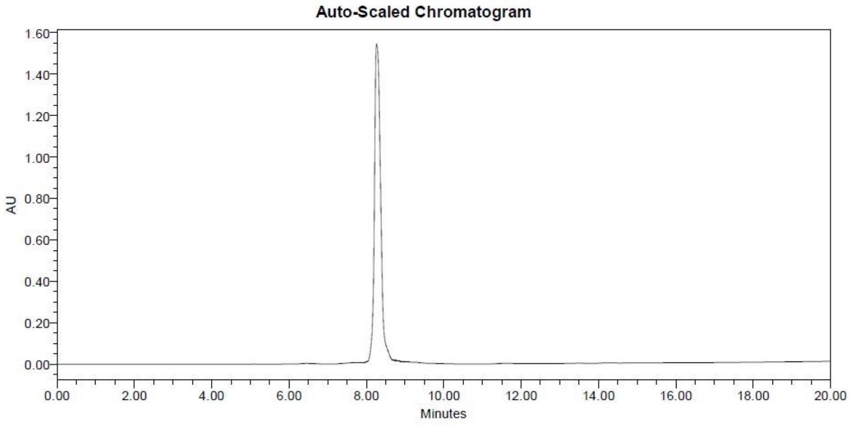


**Figure S7.** HPLC profile of the purified peptidomimetic **4**.

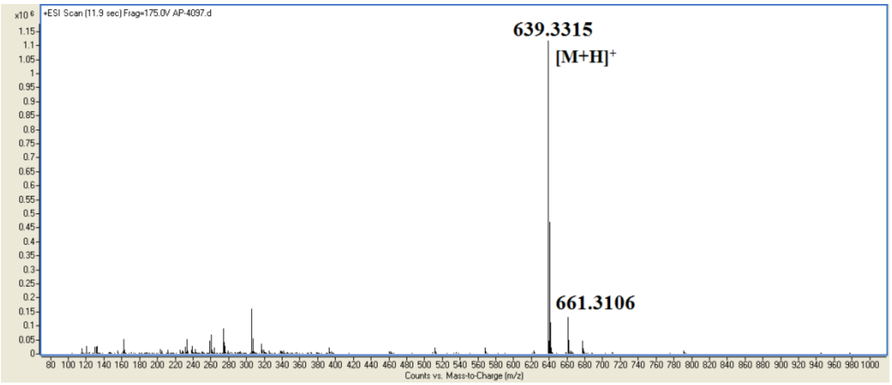


**Figure S8.** Mass spectrum of peptidomimetic **4**. Calculated mass for $C_{31}H_{43}N_8O_7$ is 639.3255 $[M+H]^+$, observed 639.3315 $[M+H]^+$ and calculated mass for $C_{31}H_{42}N_8O_7Na$ is 661.3074 [M+Na]+, observed 661.3106 $[M+Na]^+$.

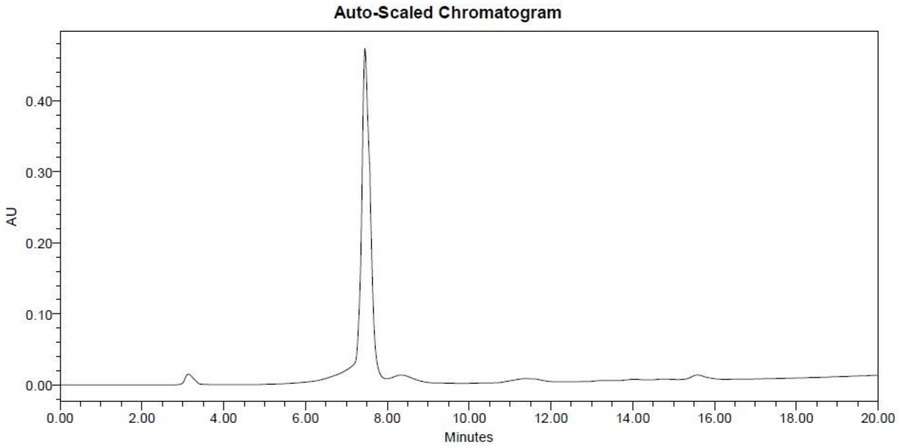


**Figure S9.** HPLC profile of the purified peptidomimetic **5**.

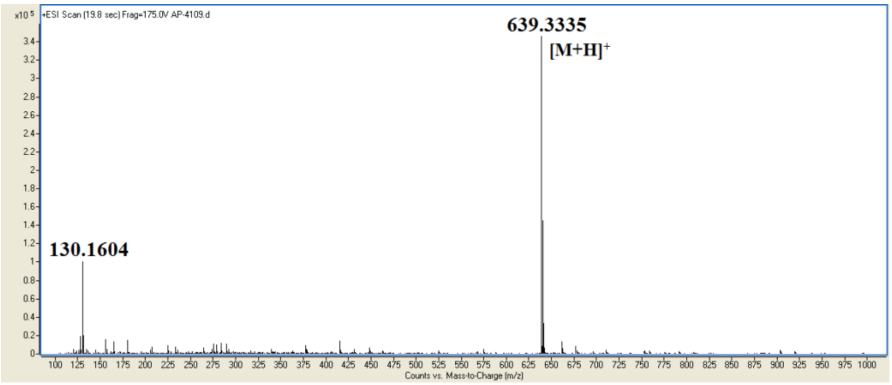


**Figure S10.** Mass spectrum of peptidomimetic **5**. Calculated mass for $C_{31}H_{43}N_8O_7$ is 639.3255 $[M+H]^+$, observed 639.3335 $[M+H]^+$ .

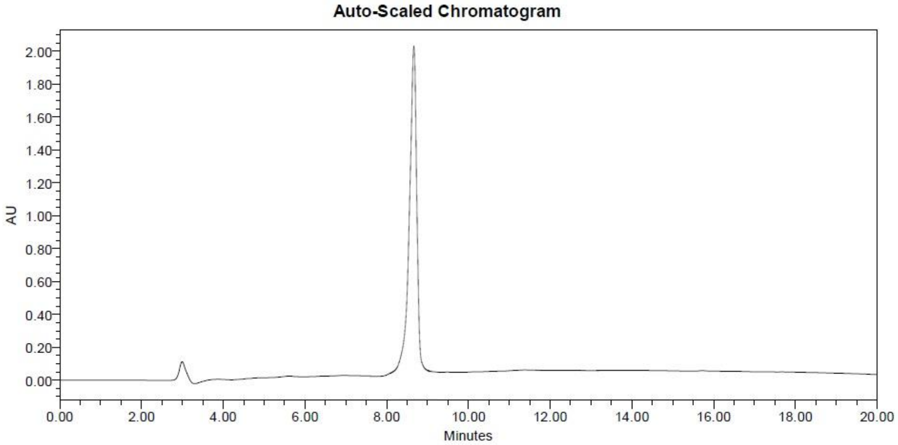


**Figure S11.** HPLC profile of the purified peptidomimetic **6**.

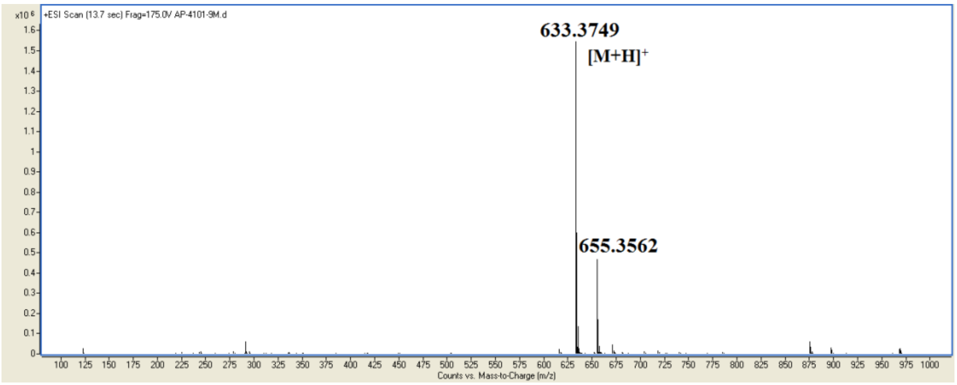


**Figure S12.** Mass spectrum of peptidomimetic **6**, Calculated mass for $C_{30}H_{49}N_8O_7$ is 633.3724 $[M+H]^+$, observed 633.3749 $[M+H]^+$ and calculated mass for $C_{30}H_{48}N_8O_7Na$ is 655.3544 [M+Na]+, observed 655.3562 $[M+Na]^+$.

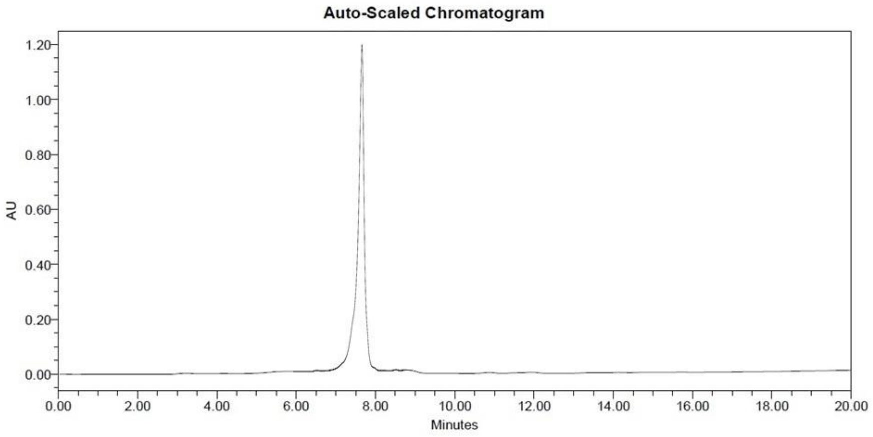


**Figure S13.** HPLC profile of the purified peptidomimetic **7**.

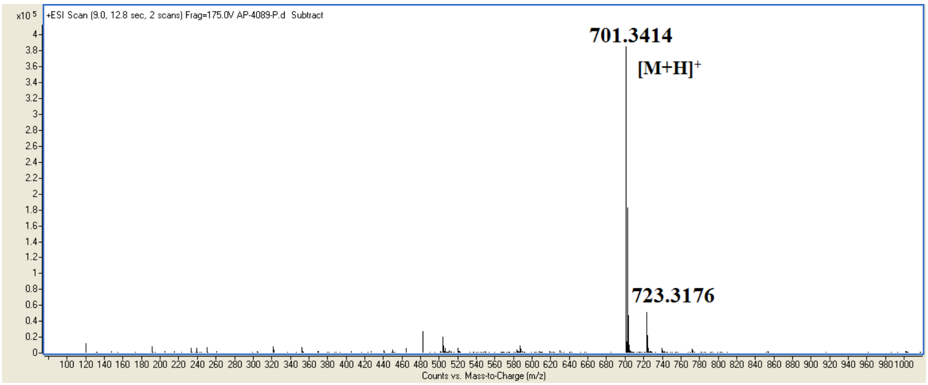


**Figure S14.** Mass spectrum of peptidomimetic **7**. Calculated mass for $C_{36}H_{45}N_8O_7$ is 701.3411 $[M+H]^+$, observed 701.3414 $[M+H]^+$ and calculated mass for $C_{36}H_{44}N_8O_7Na$ is 723.3231 $[M+Na]^+$, observed 723.3176 $[M+Na]^+$.

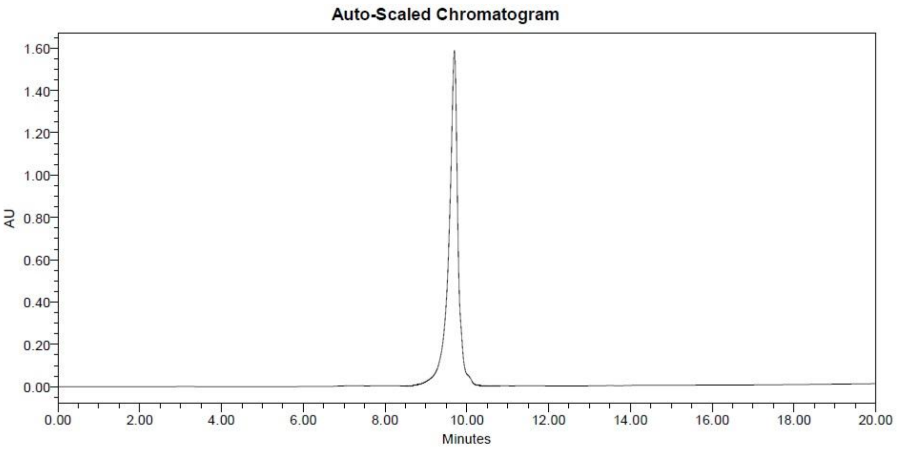


**Figure S15.** HPLC profile of the purified peptidomimetic **8**.

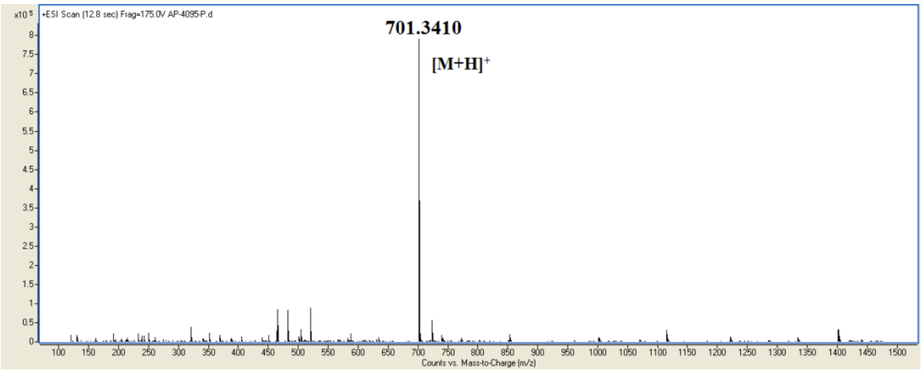


**Figure S16.** Mass spectrum of peptidomimetic **8**. Calculated mass for $C_{36}H_{45}N_8O_7$ is 701.3411 $[M+H]^+$, observed 701.3410 $[M+H]^+$.

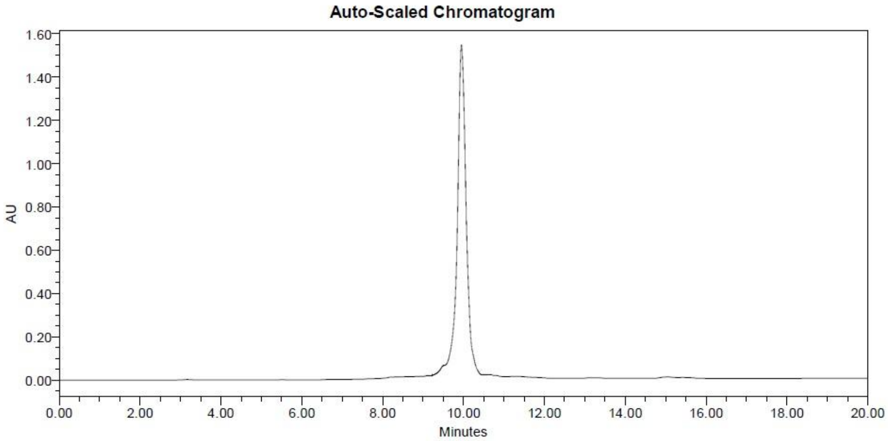


**Figure S17.** HPLC profile of the purified peptidomimetic **9**.

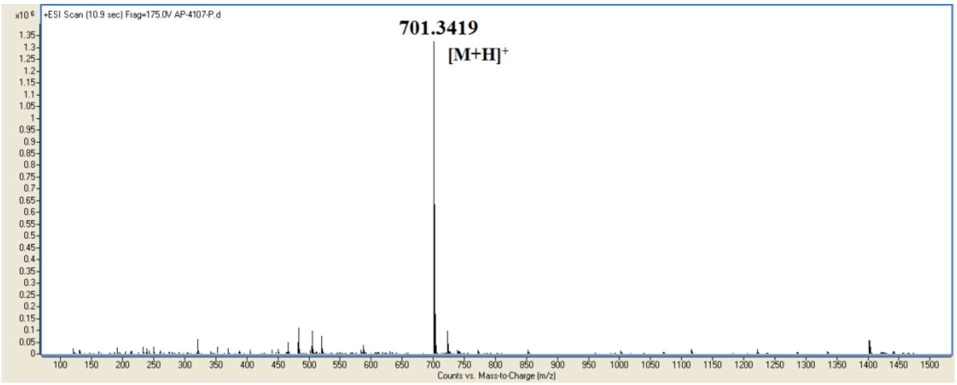


**Figure S18.** Mass spectrum of peptidomimetic **9**. Calculated mass for $C_{36}H_{45}N_8O_7$ is 701.3411 $[M+H]^+$, observed 701.3419 $[M+H]^+$.

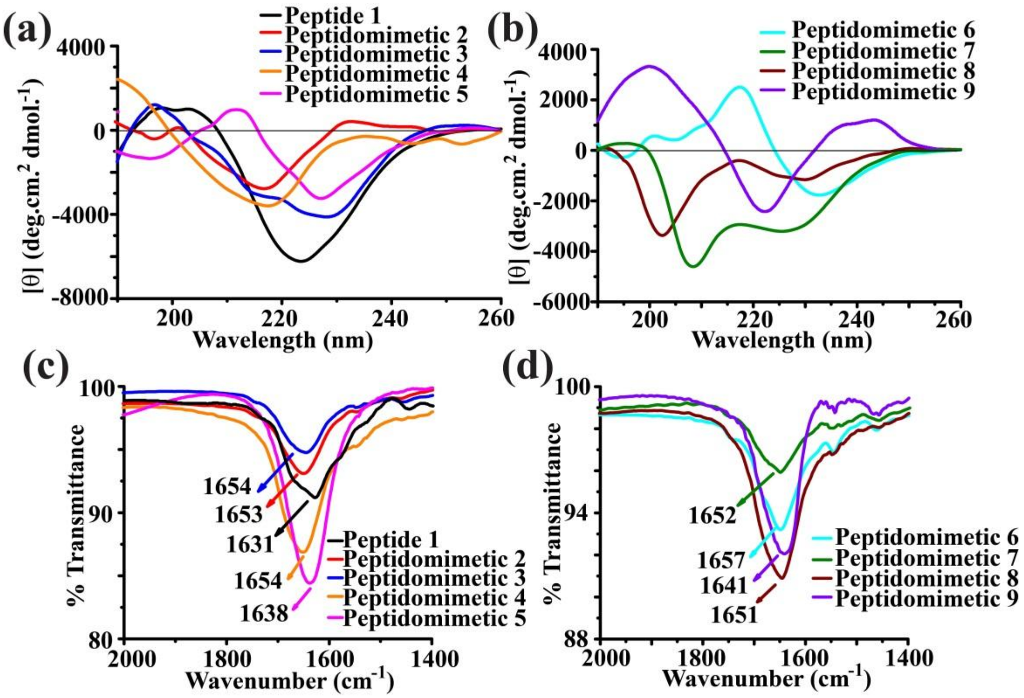


**Figure S19.** (a) & (b) CD spectra of peptide **1-9** and (c) & (d) FTIR spectra of peptide **1-9** respectively. Spectra were taken after 5 days of incubation of the peptides in PBS pH 7.4 at 37 °C.

# Inhibition of fibril formation of hIAPP by BSBHps:

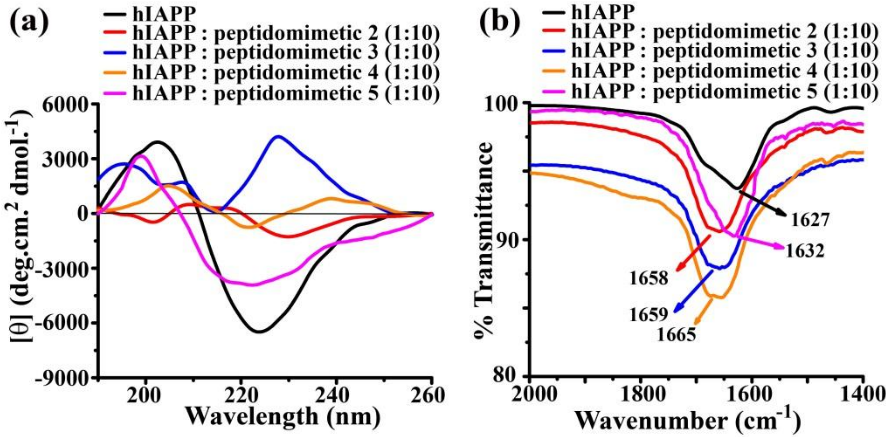


**Figure S20.** (a) CD spectra of hIAPP alone (black), and in presence of 10 fold molar excess of peptidomimetic **2** (red), **3** (blue), **4** (orange) and **5** (magenta). (b) FTIR spectra of hIAPP alone (black), and in presence of 10 fold molar excess of peptidomimetic **2** (red), **3** (blue), **4** (orange) and **5** (magenta). The spectra were recorded after 7 days of incubation at 37 °C. The concentration of hIAPP was 40 µM in PBS pH 7.4 (50 mM).

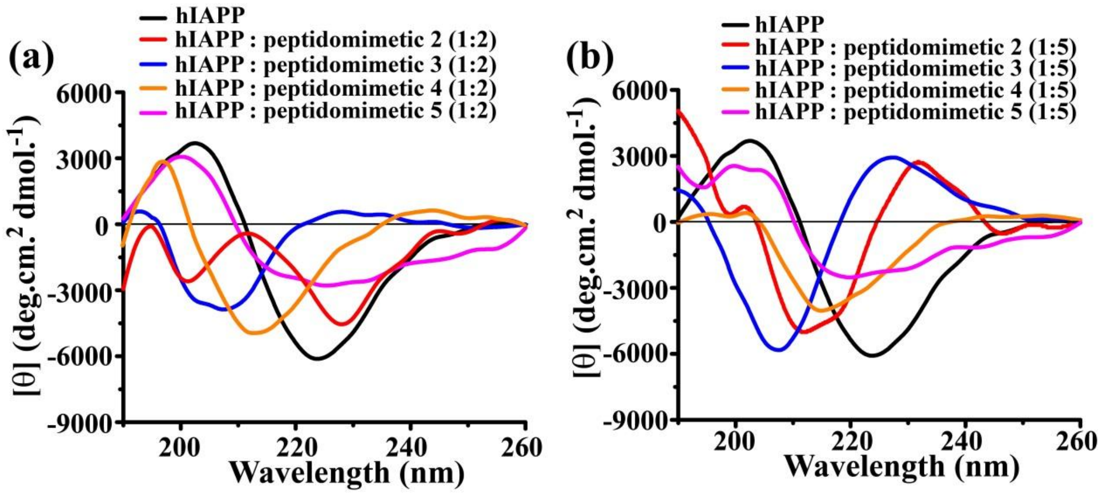


**Figure S21.** (a) CD spectra of hIAPP alone (black), and in presence of 2 fold molar excess of peptidomimetic **2** (red), **3** (blue), **4** (orange) and **5** (magenta). (b) CD spectra of hIAPP alone (black), and in presence of 5 fold molar excess of peptidomimetic **2** (red), **3** (blue), **4** (orange) and **5** (magenta). The spectra were recorded after 7 days of incubation at 37 °C. The concentration of hIAPP was 40 µM in PBS pH 7.4 (50 mM).

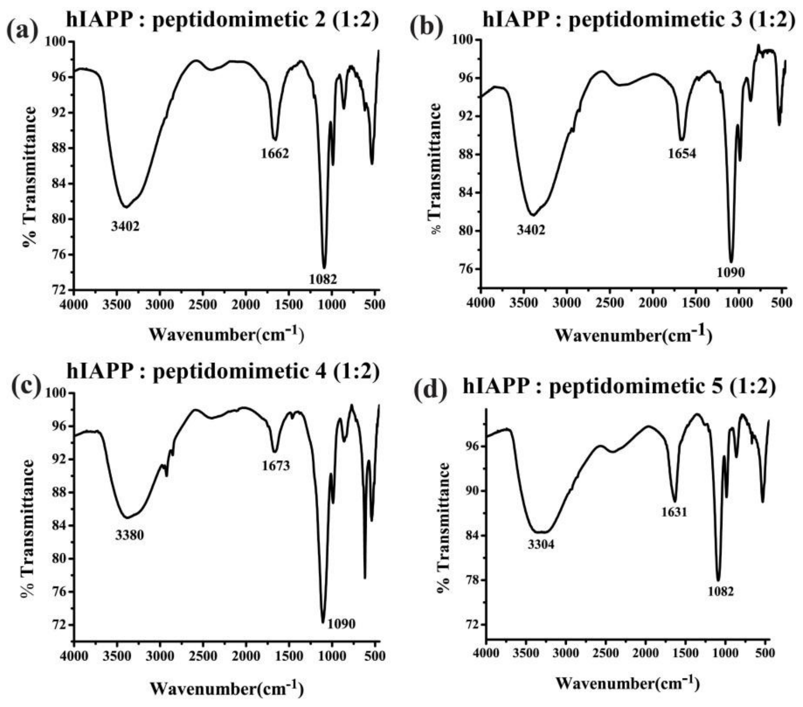


**Figure S22.** FTIR spectra of hIAPP in presence of 2 fold molar excess of peptidomimetic (a) **2,** (b) **3,** (c) **4** and (d) **5**. The spectra were recorded after 7 days of incubation at 37 °C in PBS pH 7.4 (50 mM).

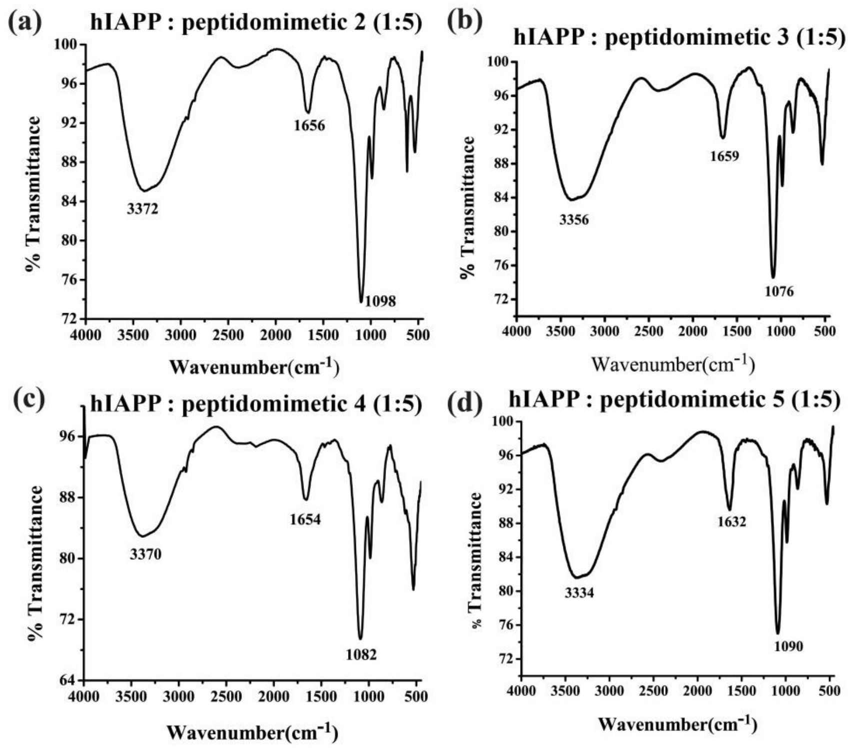


**Figure S23.** FTIR spectra of hIAPP in presence of 5 fold molar excess of peptidomimetic (a) **2,** (b) **3,** (c) **4** and (d) **5**. The spectra were recorded after 7 days of incubation at 37 °C in PBS pH 7.4 (50 mM).

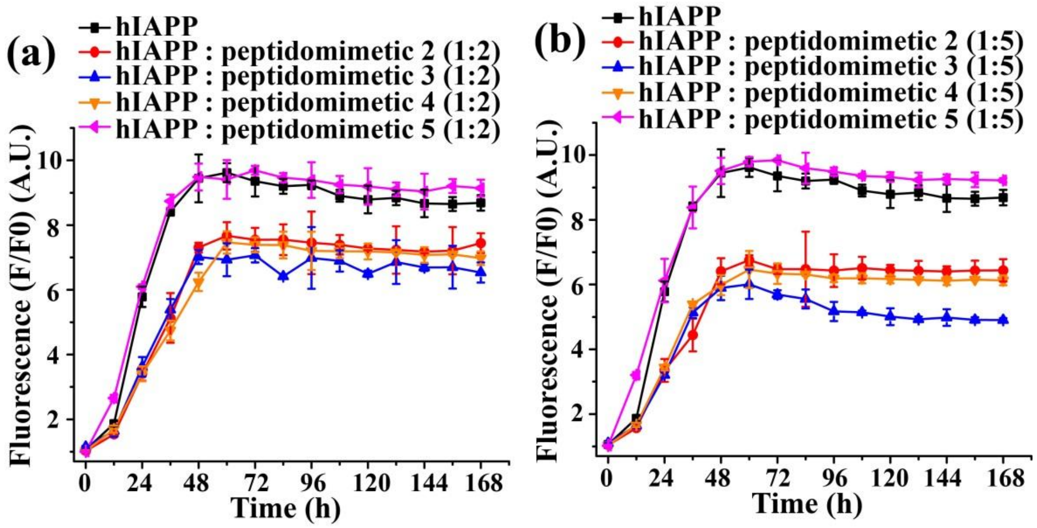


**Figure S24.** Time dependent ThT fluorescence assay of hIAPP (40µM) in absence (black) and presence of (a) 2 fold molar excess and (b) 5 fold molar excess of peptidomimetic **2** (red), **3** (blue), **4** (orange) and **5**(magenta).

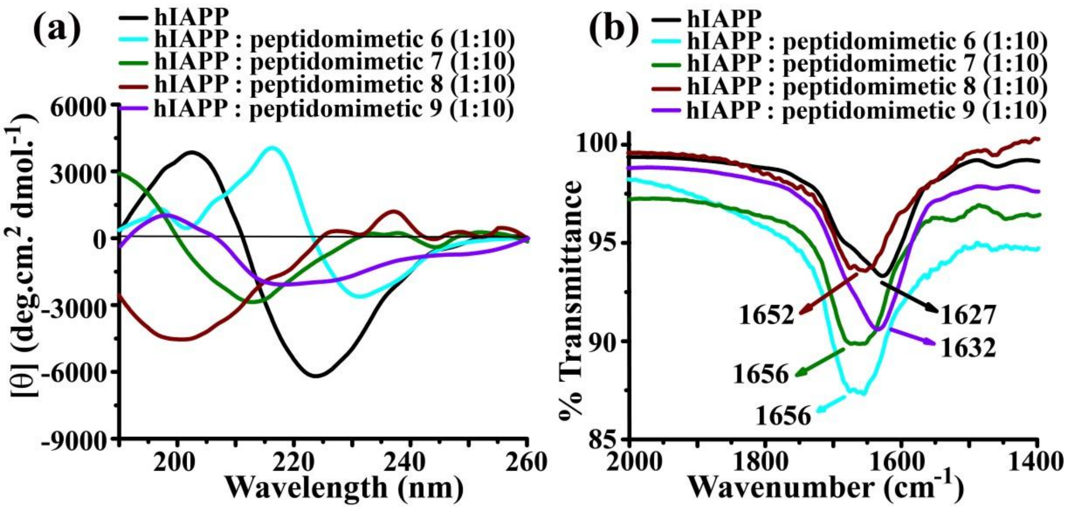


**Figure S25.** (a) CD spectra of hIAPP alone (black), and in presence of 10 fold molar excess of peptidomimetic **6** (cyan), **7** (olive), **8** (wine) and **9** (violet). (b) FTIR spectra of hIAPP alone (black), and in presence of 10 fold molar excess of peptidomimetic **6** (cyan), **7** (olive), **8** (wine) and **9** (violet). The spectra were recorded after 7 days of incubation at 37 °C. The concentration of hIAPP was 40 µM in PBS pH 7.4 (50 mM).

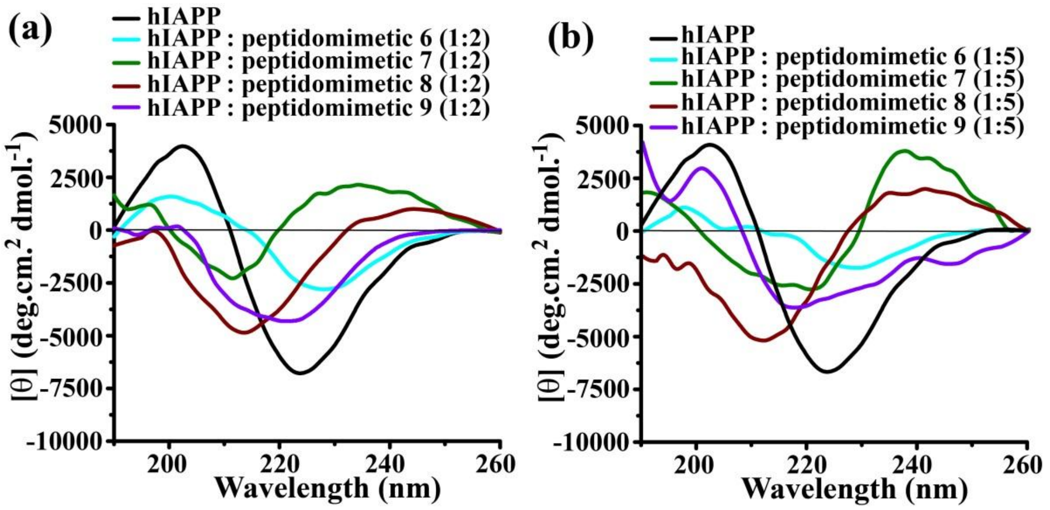


**Figure S26.** (a) CD spectra of hIAPP alone (black), and in presence of 2 fold molar excess of peptidomimetic **6** (cyan), **7** (olive), **8** (wine) and **9** (violet). (b) CD spectra of hIAPP alone (black), and in presence of 5 fold molar excess of peptidomimetic **6** (cyan), **7** (olive), **8** (wine) and **9** (violet). The spectra were recorded after 7 days of incubation at 37 °C. The concentration of hIAPP was 40 µM in PBS pH 7.4 (50 mM).

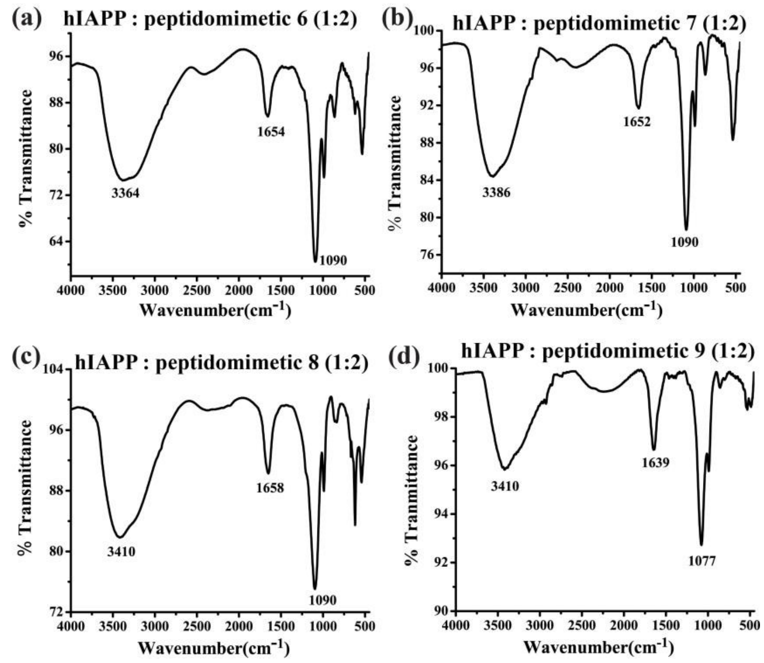


**Figure S27.** FTIR spectra of hIAPP in presence of 2 fold molar excess of peptidomimetic (a) **6,** (b) **7,** (c) **8** and (d) **9**. The spectra were recorded after 7 days of incubation at 37 °C in PBS pH 7.4 (50 mM).

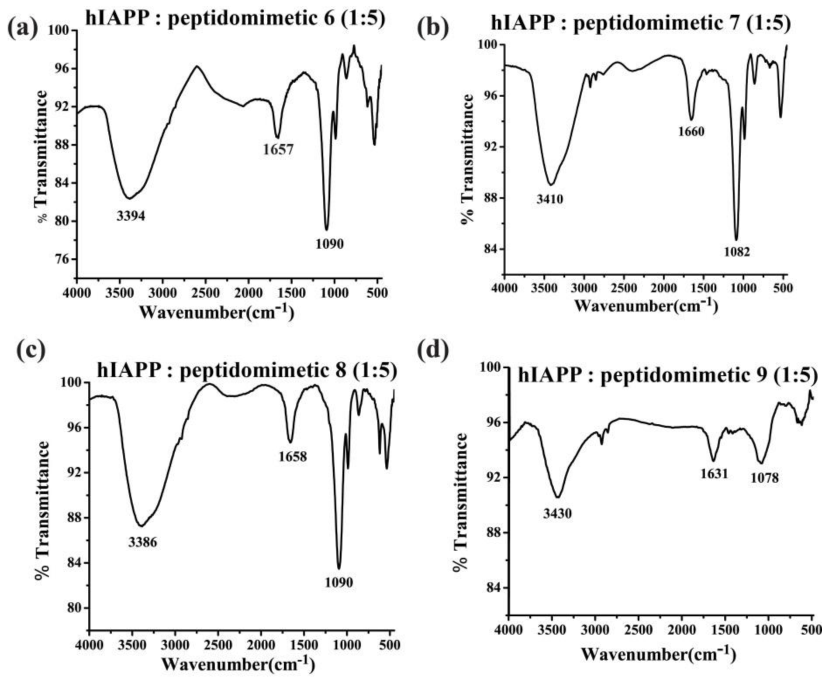


**Figure S28.** FTIR spectra of hIAPP in presence of 5 fold molar excess of peptidomimetic (a) **6,** (b) **7,** (c) **8** and (d) **9**. The spectra were recorded after 7 days of incubation at 37 °C in PBS pH 7.4 (50 mM).

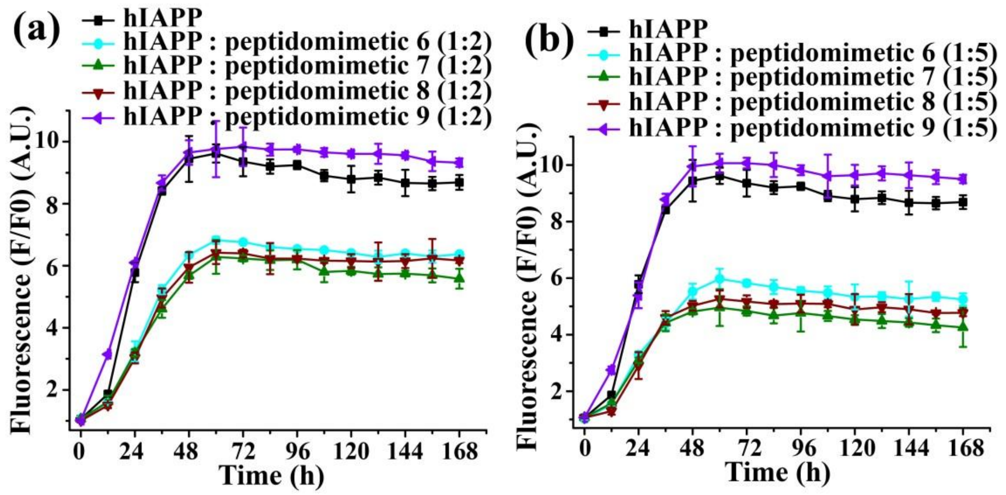


**Figure S29.** Time dependent ThT fluorescence assay of hIAPP (40μM) in absence (black) and presence of (a) 2 fold molar excess and (b) 5 fold molar excess of peptidomimetic **6** (cyan), **7** (olive), **8** (wine) and **9** (violet).

## Disruption of Preformed Amyloid Fibril of hIAPP by BSBHps:

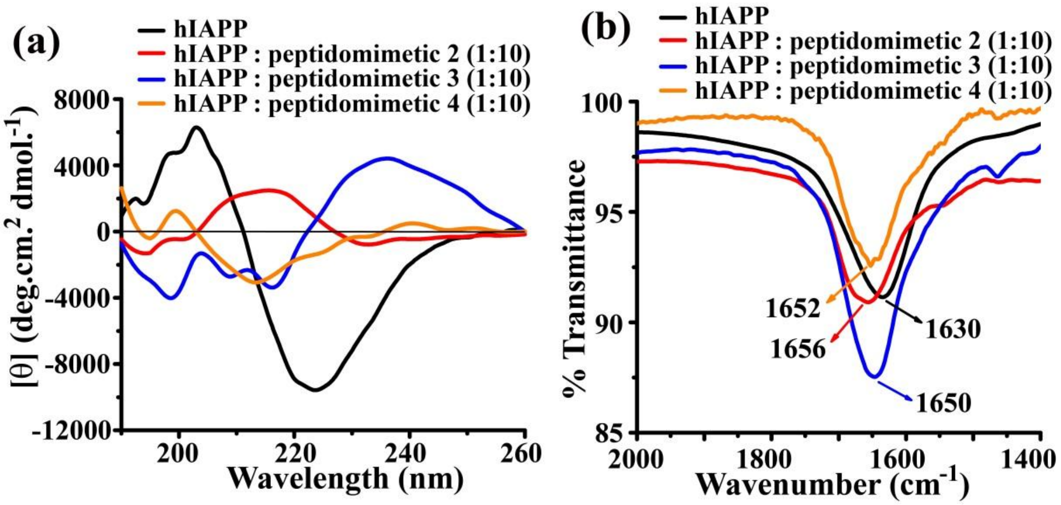


**Figure S30.** (a) CD spectra of hIAPP alone (black), and in presence of 10 fold molar excess of peptidomimetic **2** (red), **3** (blue) and **4** (orange). (b) FTIR spectra of hIAPP alone (black), and in presence of 10 fold molar excess of peptidomimetic **2** (red), **3** (blue) and **4** (orange). The spectra were recorded after 7 (2+5) days of incubation at 37 °C. The concentration of hIAPP was 40 μM in PBS pH 7.4 (50 mM).

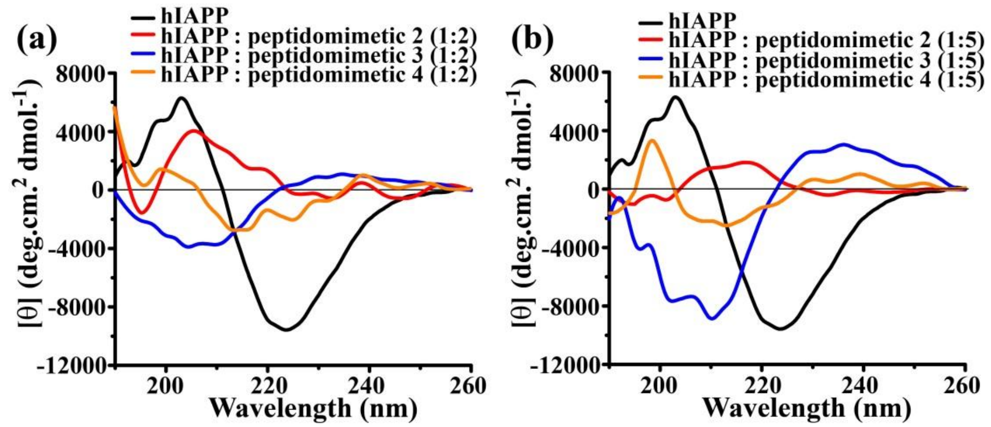


**Figure S31.** (a) CD spectra of hIAPP alone (black), and in presence of 2 fold molar excess of peptidomimetic **2** (red), **3** (blue) and **4** (orange). (b) CD spectra of hIAPP alone (black), and in presence of 5 fold molar excess of peptidomimetic **2** (red), **3** (blue) and **4** (orange). The spectra were recorded after 7 days of incubation at 37 °C. The concentration of hIAPP was 40 µM in PBS pH 7.4 (50 mM).

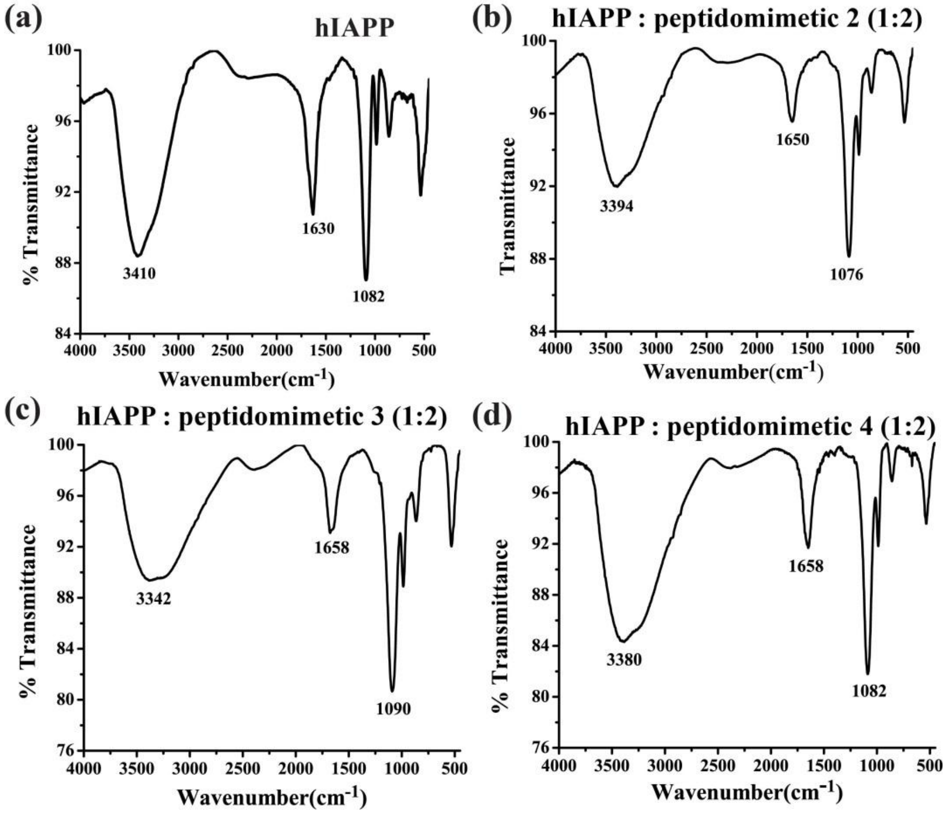


**Figure S32.** FTIR spectra of hIAPP (a) alone and in presence of 2 fold molar excess of peptidomimetic (b) **2,** (c) **3** and (d) **4**. The spectra were recorded after 7 days of incubation at 37 °C in PBS pH 7.4 (50 mM).

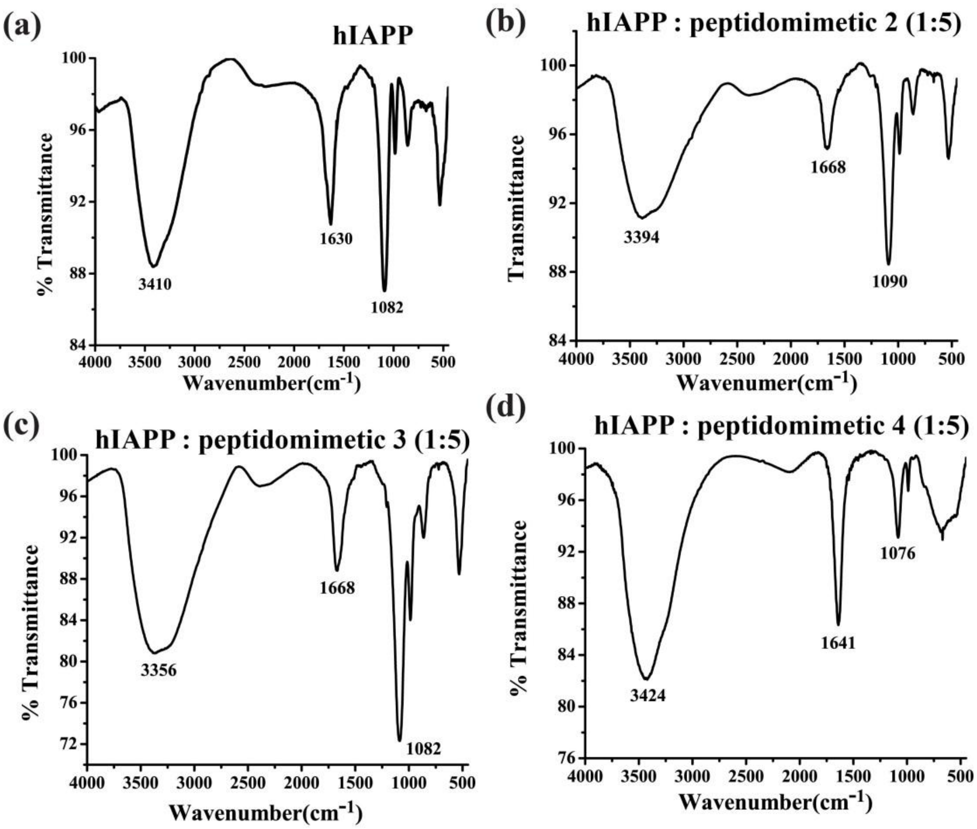


**Figure S33.** FTIR spectra of hIAPP (a) alone and in presence of 5 fold molar excess of peptidomimetic (b) **2,** (c) **3** and (d) **4**. The spectra were recorded after 7 days of incubation at 37 °C in PBS pH 7.4 (50 mM).

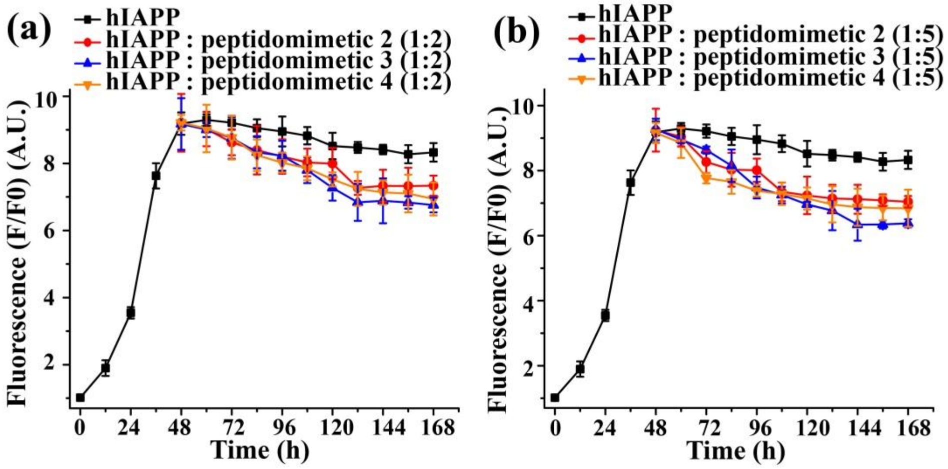


**Figure S34.** Time dependent ThT fluorescence assay of hIAPP (40μM) in absence (black) and presence of (a) 2-fold molar excess and (b) 5 fold molar excess of peptidomimetic **2** (red), **3** (blue) and **4** (orange).

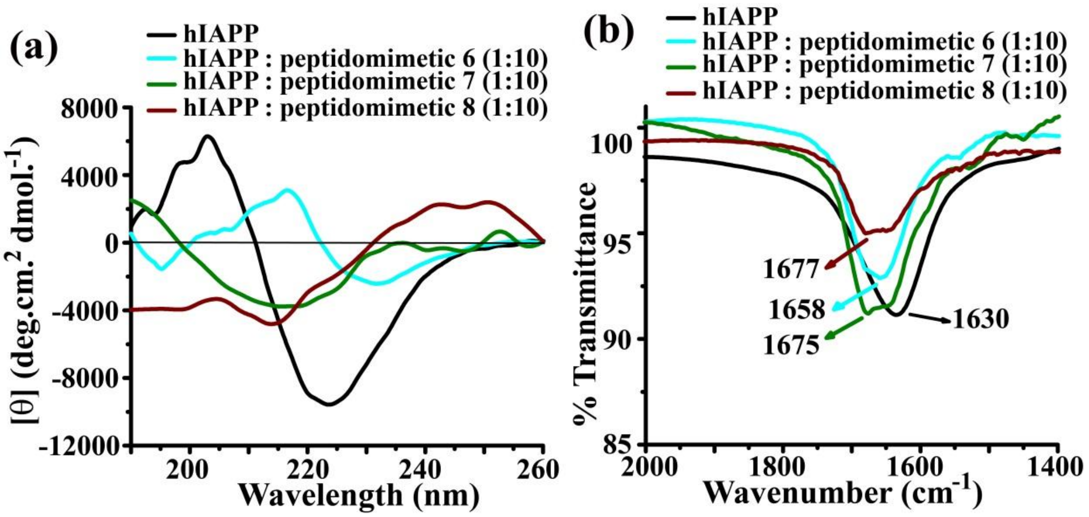


**Figure 35.** (a) CD spectra of hIAPP alone (black), and in presence of 10 fold molar excess of peptidomimetic **6** (cyan), **7** (olive) and **8** (wine). (b) FTIR spectra of hIAPP alone (black), and in presence of 10-fold molar excess of peptidomimetic **6** (cyan), **7** (olive) and **8** (wine). The spectra were recorded.

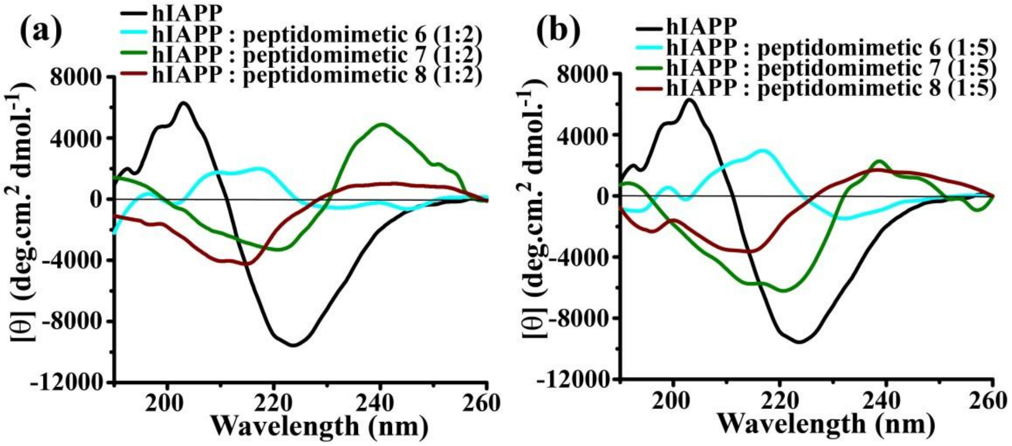


**Figure S36**. (a) CD spectra of hIAPP alone (black), and in presence of 2 fold molar excess of peptidomimetic **6** (cyan), **7** (olive) and **8** (wine). (b) CD spectra of hIAPP alone (black), and in presence of 5 fold molar excess of peptidomimetic **6** (cyan), **7** (olive) and **8** (wine).. The spectra were recorded after 7 days of incubation at 37 °C. The concentration of hIAPP was 40 µM in PBS pH 7.4 (50 mM).

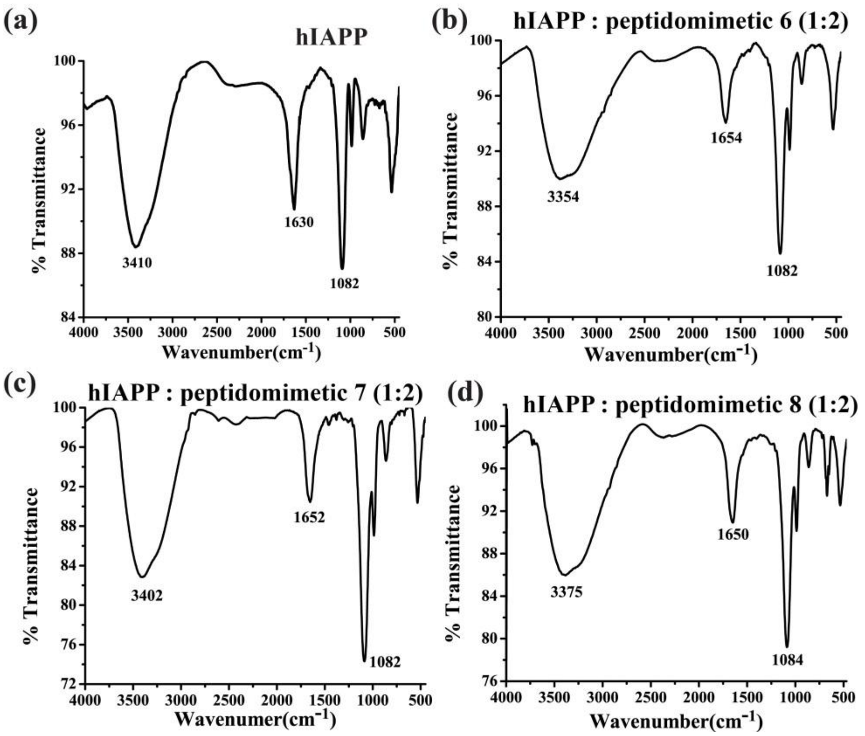


**Figure S37.** FTIR spectra of hIAPP (a) alone and in presence of 2 fold molar excess of peptidomimetic (b) **6,** (c) **7** and (d) **8**. The spectra were recorded after 7 days of incubation at 37 °C in PBS pH 7.4 (50 mM).

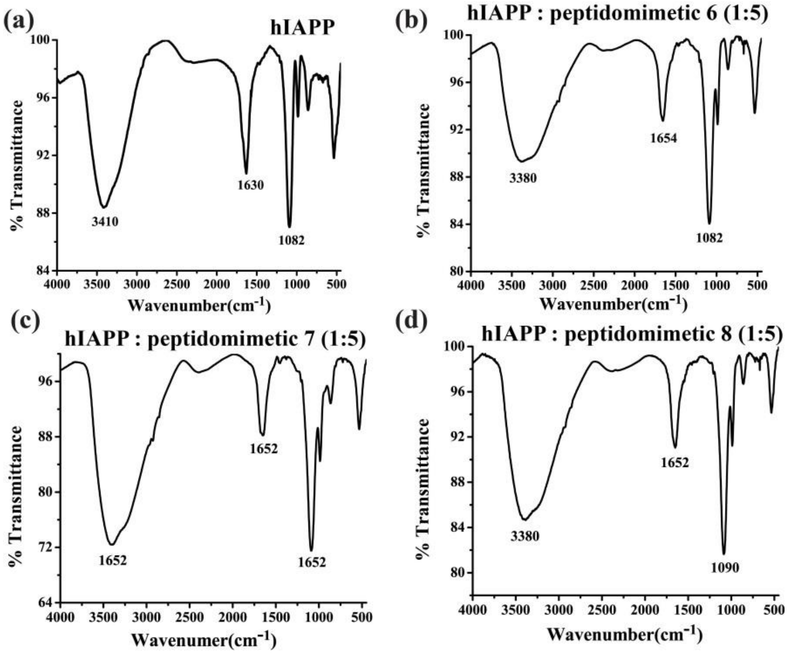


**Figure S38.** FTIR spectra of hIAPP (a) alone and in presence of 5 fold molar excess of peptidomimetic (b) **6,** (c) **7** and (d) **8**. The spectra were recorded after 7 days of incubation at 37 °C in PBS pH 7.4 (50 mM).

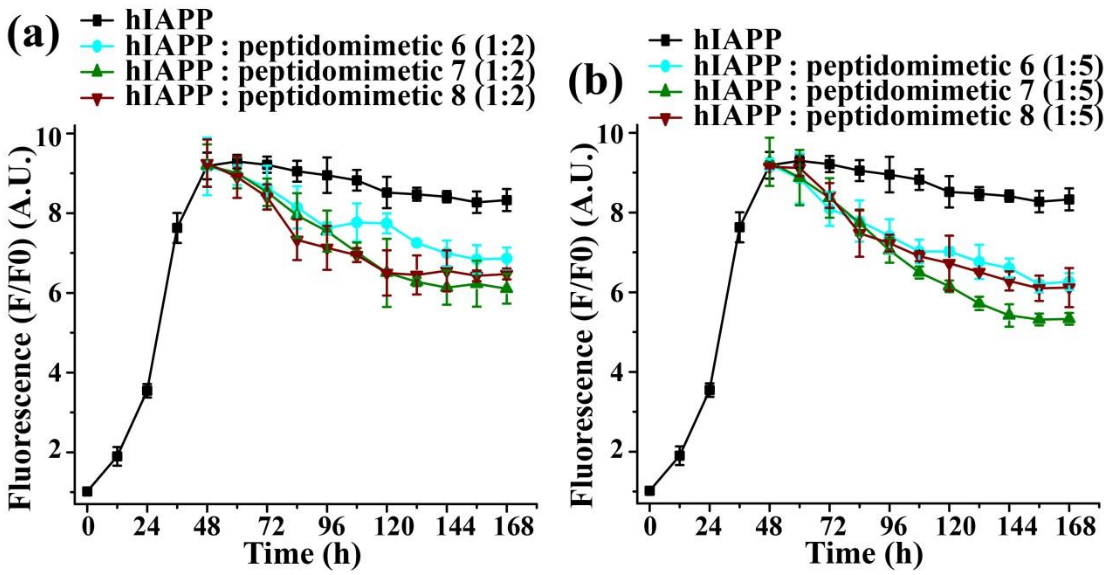


**Figure S39.** Time dependent ThT fluorescence assay of hIAPP (40μM) in absence (black) and presence of (a) 2 fold molar excess and (b) 5 fold molar excess of peptidomimetic **6** (cyan), peptidomimetic **7** (olive) and peptidomimetic **8** (wine).

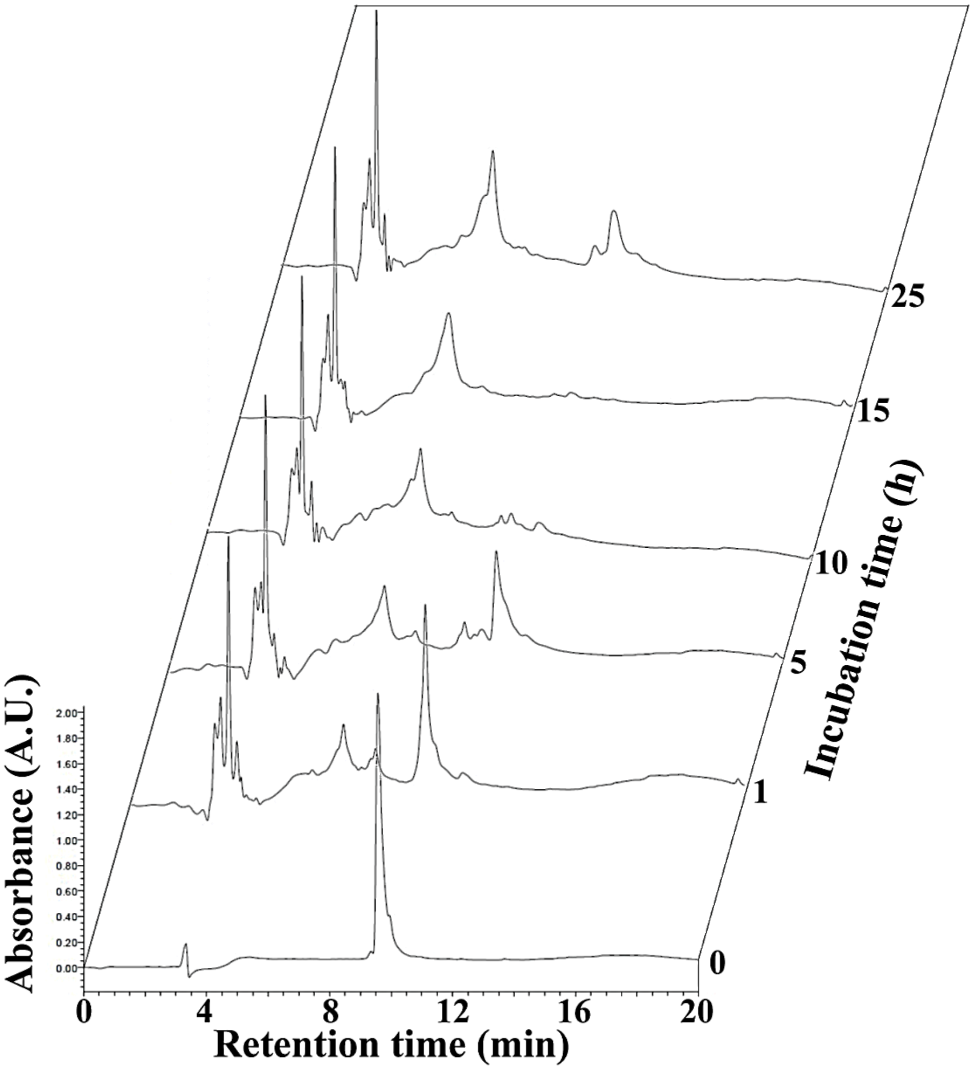


**Figure S40:** Stability kinetics of the peptide **1** in presence of proteolytic enzyme (human serum) monitored by HPLC.

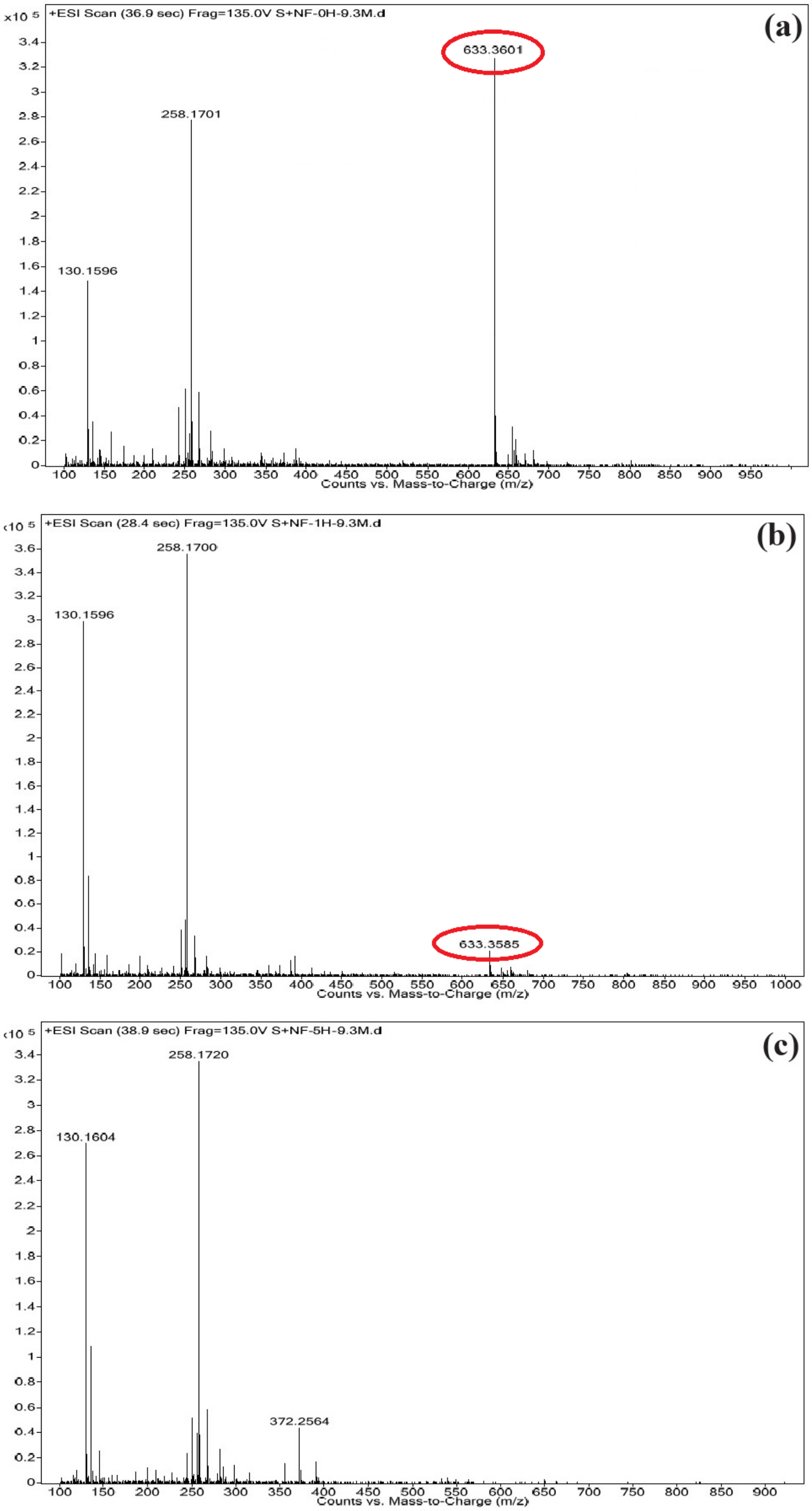


**Figure S41:** Mass spectra of peptide **1** after addition of human serum at (a) 0h, (b) 1h and (c) 5h. Calculated mass for $C_{30}H_{49}N_8O_7$ is 633.3724 $[M+H]^+$.

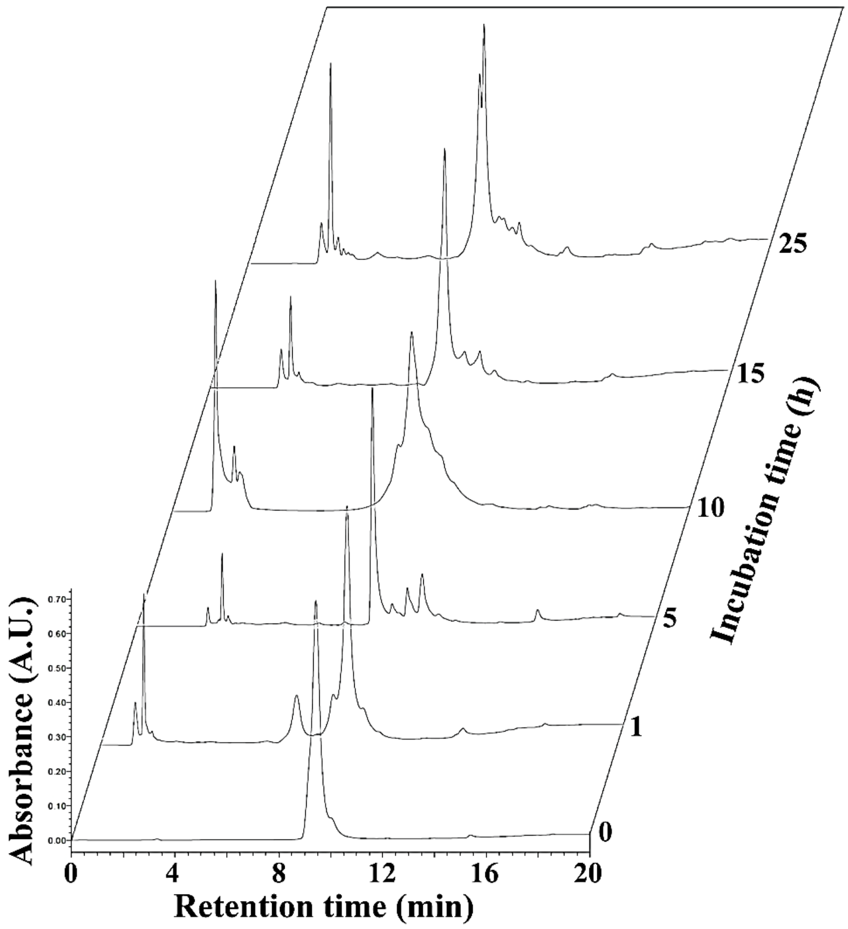


**Figure S42:** Stability kinetics of the peptidomimetic **3** in presence of proteolytic enzyme (human serum) monitored by HPLC.

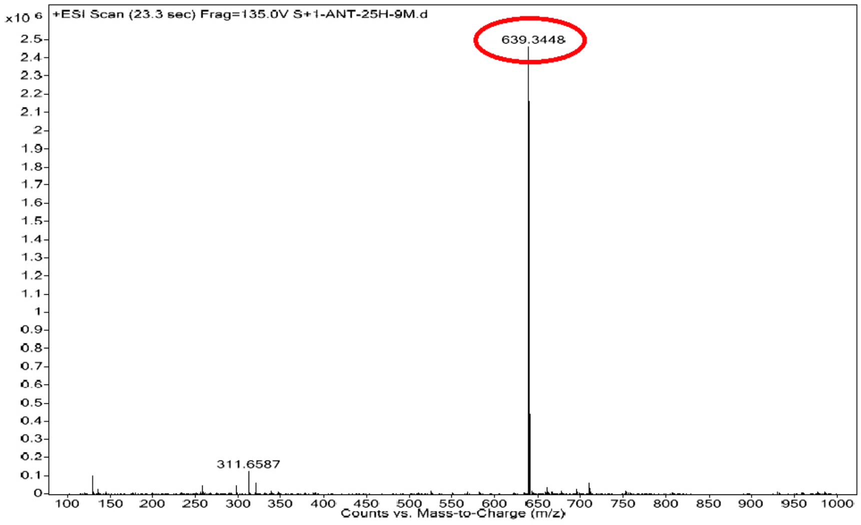


**Figure S43:** Mass spectra of peptidomimetic **3** after addition of human serum at 25h. Calculated mass $C_{31}H_{43}N_8O_7$ is 639.3255 $[M+H]^+$ and observed 639.3448$[M+H]^+$.

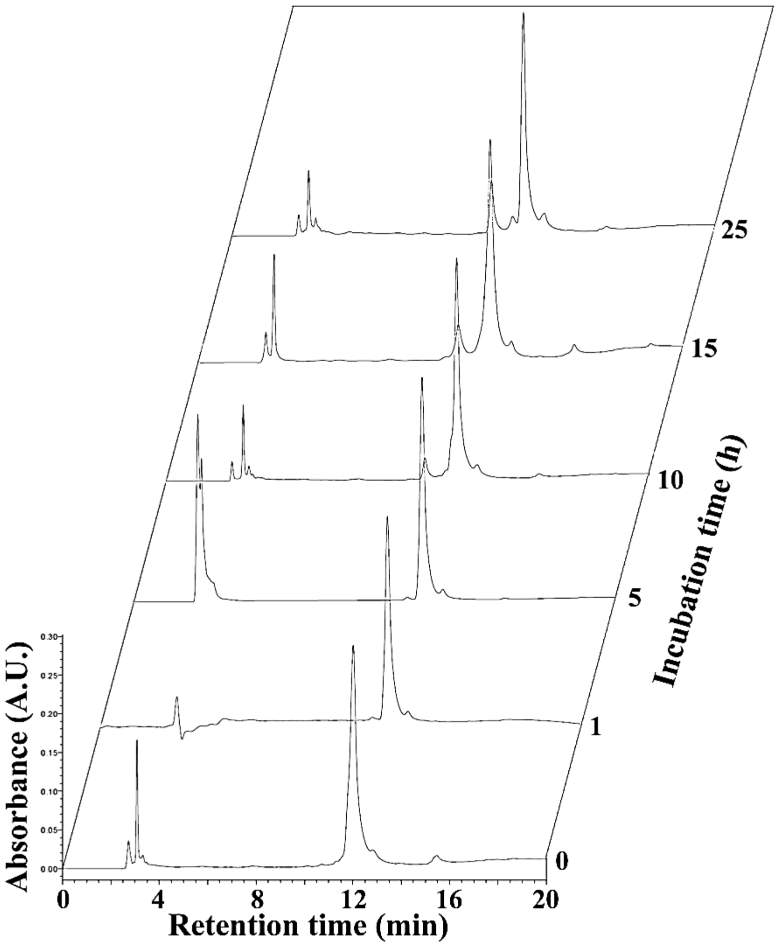


**Figure S44:** Stability kinetics of the peptidomimetic **7** in presence of proteolytic enzyme (human serum) monitored by HPLC.

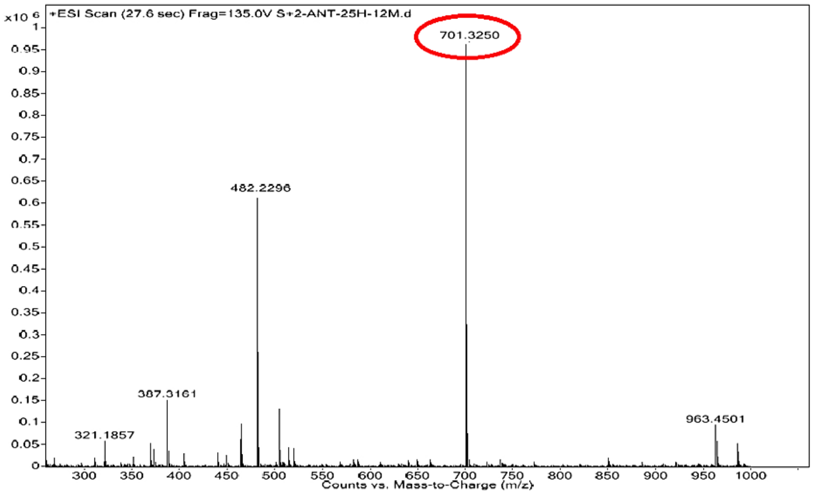


**Figure S45:** Mass spectra of peptidomimetic **7** after addition of human serum at 25h. Calculated mass $C_{36}H_{45}N_8O_7$ is 701.3411 $[M+H]^+$, observed 701.3250 $[M+H]^+$.
